# MLKL‒OPTN axis regulates herpesvirus‐induced neurological sequelae

**DOI:** 10.1002/ctm2.70353

**Published:** 2025-06-09

**Authors:** Ilina Bhattacharya, Rashmi Kadam, Tejabhiram Yadavalli, Chandrashekhar D. Patil, Hemant Borase, Ipsita Volety, Sergey Kalinin, Douglas L. Feinstein, Henry C. Tseng, Deepak Shukla

**Affiliations:** ^1^ Department of Ophthalmology and Visual Sciences University of Illinois Chicago Chicago Illinois USA; ^2^ Department of Microbiology and Immunology University of Illinois Chicago Chicago Illinois USA; ^3^ Department of Pathology University of Illinois Chicago Chicago Illinois USA; ^4^ Department of Psychiatry University of Illinois Chicago Chicago Illinois USA; ^5^ Duke Eye Center and Department of Ophthalmology Duke University Medical Center Durham North Carolina USA

**Keywords:** cell death, demyelinating disorders, HSV‐1, MLKL, OPTN

## Abstract

**Background:**

Herpes simplex virus‐1 (HSV‐1) infections are lifelong and linked to neurological diseases such as multiple sclerosis (MS), yet the underlying mechanisms in the host remain poorly understood.

**Methods and Results:**

This study investigates new molecular dynamics following HSV‐1 infection, uncovering the pivotal role of the mixed lineage kinase domain‐like (MLKL) protein. Beyond its known function in necroptosis, MLKL was found to control HSV‐1 transport into the nucleus, tightly regulated by Optineurin (OPTN). We evidenced an essential regulatory interaction between MLKL and OPTN, governing MLKL's activity in both necroptosis‐dependent and independent pathways. In vivo, studies using Optn knockout mice demonstrated how this MLKL‐OPTN axis contributes to demyelination and neurological symptoms mimicking MS. This axis critically prevents oligodendrocyte death and the associated demyelination during HSV‐1 infection. Furthermore, pharmacological interventions with Necrosulfonamide (NSA), an MLKL inhibitor, showed therapeutic potential in preserving myelin integrity and reducing neurological deficits in HSV‐1‐infected models, suggesting a viable strategy for managing virus‐induced neurodegeneration.

**Conclusion:**

Our findings highlight the significant role of MLKL in HSV‐1 pathogenesis and suggest that MLKL dysregulation is a key mechanism behind severe neurological damage.

**Key points:**

- MLKL plays a significant role in regulating endosomal transport of HSV‐1 to nucleus during early stages of infection.
- Formation of p‐MLKL bodies during HSV‐1 infection leads to death of oligodendrocyte and subsequent demyelination.
- OPTN can negatively modulate MLKL levels to restrict infection and consequential oligodendrocyte death during HSV‐1 infection.

## Graphical abstract

1. MLKL plays a significant role in regulating endosomal transport of HSV‐1 to nucleus during early stages of infection.
2. Formation of p‐MLKL bodies during HSV‐1 infection leads to death of oligodendrocyte and subsequent demyelination.
3. OPTN can negatively modulate MLKL levels to restrict infection and consequential oligodendrocyte death during HSV‐1 infection.

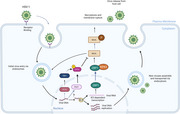

## 1 INTRODUCTION

The ubiquitous presence of herpesviruses in nature predicates a significant impact on human health, often resulting in enduring infections with consequential lifelong health complications and neurological ramifications.^1^ Among these, herpes simplex virus‐1 (HSV‐1), serving as a prototypical member of the herpesvirus family, establishes persistent latency within sensory neurons after primary infection at orofacial and ocular mucosal surfaces.^2^, ^3^ Despite the widespread prevalence of HSV‐1, overt clinical manifestations may arise infrequently, yet the enduring implications of lifelong infections remain unknown, necessitating the need for new knowledge on the underlying pathogenic mechanisms that foster inflammatory responses, cell death and neurodegenerative sequelae following HSV‐1 infection.^4^

Our laboratory previously identified Optineurin (OPTN) as a host antiviral restriction factor that restricts HSV‐1 replication and spread.^5^ We also demonstrated that cells deficient in OPTN are more susceptible to necroptotic cell death following HSV‐1 infection.^5^ Necroptosis, an intricately regulated programmed cell death mechanism, is orchestrated by the coordinated action of receptor‐interacting protein kinase 3 (RIPK3) and its downstream mediator, mixed lineage kinase domain‐like (MLKL).^6^ RIPK3 is activated via either receptor‐interacting protein kinase 1 (RIPK1) or Z‐nucleic acid‐binding protein 1 (ZBP1).^7^ Activated RIPK3 phosphorylates MLKL leading to its oligomerisation.^8^ This oligomerisation triggers the translocation of MLKL to the plasma membrane to execute permeabilisation of the membrane.^9^, ^10^ Building on this understanding of necroptotic signalling, we sought to investigate whether OPTN plays a direct role in modulating components of this pathway beyond its established antiviral functions.

In our study, we identify a non‐canonical regulation of MLKL aided by OPTN. Recent studies have unveiled the role of OPTN, an autophagy adaptor protein, in facilitating the lysosomal degradation of key HSV‐1 proteins such as glycoprotein B (gB) and VP16, thereby attenuating the viral burden.^5^ Loss of functional mutations within the OPTN gene has been linked to chronic inflammatory and neurodegenerative pathologies; however, the precise mechanisms and factors governing its involvement in such pathological signalling are poorly understood.^11^ Similarly, any connection between OPTN's regulation of MLKL functions and the consequence of this regulation remains unknown. Our study unveils a novel regulatory axis governing MLKL functionality surpassing necroptotic activation of MLKL during HSV‐1 infection. To address this, we knocked out OPTN in RIPK3 expressing cell line (human corneal epithelial [HCE] cell line), which is also a target cell line for HSV‐1 infection. Additionally, we used a RIPK3‐independent cell line to address OPTN's role in MLKL regulation independently of RIPK3, which subverts its canonical role in necroptosis to a non‐canonical role in endosomal trafficking pathway. We demonstrate direct binding between MLKL and OPTN, a novel discovery highlighting this critical axis. In delineating this regulatory framework, our study provides new facets to MLKL's non‐necroptotic repertoire, particularly its involvement in endosomal trafficking pathways pivotal for HSV‐1 virion transit to the nucleus, thereby inadvertently augmenting viral replication rates.^12^

Nevertheless, we have also addressed the canonical function of MLKL in the absence of OPTN in animal models. We observed the aggregation of MLKL within oligodendrocytes during HSV‐1 infection, thereby exacerbating neuronal demyelination processes in the Optn ‒/‒ model. This widespread demyelination mirrors the neuropathological alterations characteristic of multiple sclerosis (MS)‐like disorders, suggesting a potential mechanistic link between HSV‐1 infection and MS‐like pathogenesis.^13^ MS is a chronic of the central nervous system where the immune system attacks the protective myelin sheath resulting in demyelination and consecutive neurodegeneration.^14^ While the exact aetiology of MS remains elusive, both genetic predisposition and environmental factors, including viral infections, have been implicated in its pathogenesis. Notably, herpesviruses such as Epstein‒Barr virus and varicella‒zoster virus (VZV) have been strongly associated with MS onset and progression.^15^, ^16^ Studies have also attempted to link HSV‐1 to MS; however, defining a causal or contributory role has remained challenging.^17^ This study highlights a direct molecular mechanism, a previously unrecognised effector pathway, that connects HSV‐1 infection to MS‐like demyelinating pathology via dysregulation of the MLKL‒OPTN axis. Necrosulphonamide (NSA), an inhibitor of MLKL, demonstrated therapeutic promise in maintaining myelin integrity and alleviating neurological deficits in models infected with HSV‐1.^18^

Our findings uncover two new aspects of MLKL: one is a trafficking mechanism where MLKL's involvement in endocytic pathways inadvertently aids the transport of HSV‐1 virions to the nucleus; and the aggregation of MLKL within oligodendrocytes during HSV‐1 infection suggesting its role in maintaining myelin integrity and alleviating neurological deficits in models infected with HSV‐1.

## 2 METHODS

### 2.1 Cell lines, viruses and tissues

HeLa (OPTN +/+) and HeLa OPTN knockout (KO) (OPTN ‒/‒) cells were provided by Dr. Richard Youle (National Institutes of Health) and cultured in were grown in Dulbecco's Modified Eagle Media (DMEM) (Gibco) supplemented with 10% (v/v) Fetal Bovine Serum (FBS) (Gibco) and 1% (v/v) penicillin‒streptomycin (Gibco). Vero cells were obtained from ATCC. SV40‐immortalised HCE cell line was obtained from Dr. Kozaburo Hayashi (National Eye Institute). Luhmes cells were provided by Dr. David Bloom (University of Florida) and cultured as previously described.^19^

The HSV‐1 McKrae strain (HSV‐1) was provided by Dr. Homayon Ghiasi (Cedars Sinai). HSV‐1 KOS and 17 strains were provided by Dr. Patricia Spear (Northwestern University). HSV‐1 K26RFP strain was provided by Dr. Prashant Desai (Johns Hopkins University). Human brain (normal) tissue slides (cat no. GTX2203) and human brain (multiple sclerosis) tissue slides (cat no. GTX2204) were obtained from GeneTex.

### 2.2 Generation of CRISPR/Cas9 KO model

To create OPTN ‒/‒ in the HCE cell line, we used three Alt‐R CRISPR‐Cas9 crRNAs: 5′‐GTTCAGACACGATGCCCAAC‐3′, 5′‐CCTGGACACGTTTACCCCGG‐3′ and 5′‐CCAGTGGAGACTGTTCTCGT‐3′. The three guides were pooled together to target exons 3 and 5, and exon 6 to ensure complete abrogation of the protein expression. The cells were electroporated using Amaxa Nucleofector II using program T‐20. The crRNAs were incubated with TracrRNA at a 1:1 ratio (300 µM) and incubated at 95°C for 5 min. Cas9 enzyme was added to the complex of crRNA + TracrRNA at a ratio of 1:3 to form the ribonucleoprotein (RNP) complex and incubated for 10 min at room temperature (RT). After 72–96 h of nucleofection, cells were sorted for single‐cell cloning and screened for the desired clone.

### 2.3 Plasmids, antibodies and chemical reagents

The full‐length OPTN and OPTN 1–424 plasmid were provided by Dr. Beatrice Yue (University of Illinois Chicago).^20^

The following antibodies and stains were used in this study: DAPI (4',6‐diamidino‐2‐phenylindole) (D9542, Sigma), mouse monoclonal to LAMP1 (Abcam, #ab25630, [H4A3]), RIPK1 antibody (Abclonal #A7414), RIPK1 rabbit polyclonal antibody (Abclonal #A7414), RIPK3 rabbit polyclonal antibody (Abclonal #A5431), MLKL rabbit monoclonal antibody (Abclonal #A21894), phospho‐MLKL‐T357/S358/S360 rabbit polyclonal antibody (Abclonal #AP0949), epidermal growth factor receptor (EGFR) rabbit polyclonal antibody (Abclonal #A11351), mouse anti‐EEA1 monoclonal antibody, clone 14 (BD Biosciences #610456), rabbit polyclonal anti‐GAPDH (Proteintech 10494‐1‐AP), rabbit polyclonal anti‐OPTN (C‐terminal) (cayman chemical, no. 100000), goat anti‐rabbit immunoglobulin G (IgG) (H + L) highly cross‐adsorbed secondary antibody, Alexa Fluor 647 (Thermo Fisher, #A‐21245), goat anti‐mouse IgG (H + L) highly cross‐adsorbed secondary antibody horseradish peroxidase (HRP) (Thermo Fisher, 31432), goat anti‐rabbit IgG (H + L), cross‐adsorbed secondary antibody HRP (Thermo Fisher, G‐21234) and anti‐HSV1 + HSV2 gB antibody (Abcam #ab6506),

The following chemicals were used in this study: bafilomycin A1 (Sigma) to inhibit autophagic flux, cycloheximide (Sigma) to block de novo protein synthesis and recombinant human EGF (Peprotech). Lipofectamine 2000 (Thermo Fisher) and RNAiMAX (Thermo Fisher) for transfections. Necrostatin 1s (Nec1s; Selleckchem) for inhibition of necroptosis. NSA (Selleckchem) for inhibition of MLKL.

### 2.4 Mice studies

#### 2.4.1 Ocular HSV‐1 infection and treatment in mice

C57BL/6 (Optn +/+) mice, bred and housed at the university biological resource laboratory, were used. Optn ‒/‒ mice used in this study were provided by Dr. Henry Tseng (Duke University). All experiments were conducted in compliance with the regulations of the University of Illinois at Chicago. All animal procedures were conducted in accordance with institutional guidelines and approved protocols to ensure humane treatment of mice. All mice were infected with the HSV‐1 McKrae strain via corneal scarification. Corneal scarification was performed using a 30‐gauge needle, followed by the application of HSV‐1 (McKrae) to the eye. All drug treatments were intraperitoneally administered twice daily after 4 days of HSV‐1 infection.

#### 2.4.2 Experimental Autoimmune Encephalomyelitis (EAE) mice model

EAE reagents were purchased from Hooke Laboratories (Kit #EK‐2110). Ten‐week‐old female C57BL/6 mice were injected with 200 µg MOG35‐55 peptide emulsified in Complete Freund's Adjuvant (CFA) (100 µL s.c. injection done at two locations on the midline of the upper and lower back). Two hours later, mice received an i.p. injection of pertussis toxin (PT; 125 ng in 100 µL Phosphate Buffered Saline (PBS)), and then 24 h later, a second PT injection.Control ('Mock') mice were treated identically to EAE mice but the MOG peptide was omitted. (‘’): i Clinical signs were scored as: 0, no clinical signs; 1, limp tail; 2, impaired righting; 3, paresis of one hind limb; 4, paresis of two hind limbs; 5, death. Scoring was performed every other day at the same time by the same investigator blinded to allocation. At the end of the study, mice were euthanised with CO_2_, brains removed, and one‐half used to prepare proteins for Western blot analysis. Spinal cords were removed, and the lumbar area was dissected and post‐fixed in 4% paraformaldehyde for 24 h. Paraffin blocks were prepared, sectioned at 8 µm and mounted onto Superfrost Plus slides (Fished Scientific #12‐550‐15).

### 2.5 Behavioural studies

Eight‐week‐old Optn +/+ and Optn ‒/‒ mice went through all the behavioural studies listed below. These mice were then infected with HSV‐1 by corneal scarification. After 8 days of infection, the mice went through all the behavioural studies again to compare the effects of infection. Most of these behavioural studies used in this study have been adapted from previously standardised behavioural methods.

#### 2.5.1 Tape removal test

In this test,^21^ a small adhesive tape (.5 cm × .5 cm) was applied on the left forepaw of the mouse and then the mouse was placed in the testing box. Two timers were used to record the time to contact and time to remove, respectively. The time to contact is the amount of time the mouse takes to react to the tape's presence. The time to remove is defined as the time taken by the mouse to remove the tape completely. These tests help to define the sensorimotor capabilities of the mouse.

#### 2.5.2 Grip test

In this test,^22^ the animal was placed on the cage top, which was then inverted and suspended 20 cm over soft bedding; the time from the inversion to when the animal falls was recorded. The animal was removed from the apparatus and placed back into its home cage.

#### 2.5.3 Rotarod test

In this test,^23^ mice went through pretraining and testing sessions. In pretraining, the mouse was placed on the rod at an initial speed of 4 rotations per minute (rpm) for 60 s. Three pretraining sessions were performed for each animal. In the testing session used to assess the motor functions, the mouse was placed on the rod at an initial speed of 4 rpm, and then the speed was increased gradually to 44 rpm over 300 s. The testing session ended as the mouse fell off the rod. Each mouse went through three consecutive testing sessions with 10‐min intervals in between. The time spent on the rod is recorded automatically for each animal by the rotarod, and the average performance in the three consecutive trials was used for comparisons.

#### 2.5.4 Ledge test

This test^24^ helps to understand the sensorimotor balance defects in mice. In this test, the mice will be lifted from the cage and placed on the ledge of the cage (ledge is the edge of the open cage that the wire top rests upon). The ledge test score will be recorded for each mouse. The scores will be assigned according to the following criteria:

0—Normal wild‐type mice can walk along the ledge without losing their balance and can descend back into the cage gracefully using their paws.
1—If the mouse loses its footing while walking along the ledge but otherwise appears coordinated.
2—If it does not effectively use its hind legs, rather than its paws when descending into the cage.
3—If it falls off the ledge, or so, while walking or attempting to lower itself, or shakes and refuses to move at all despite encouragement.

### 2.6 Western blot

Proteins were extracted using RIPA buffer and analysed by SDS‒PAGE followed by immunoblotting with standard blocking, antibody incubation and chemiluminescent detection protocols. Detailed methodology is described in our previous publication.^5^

ImageJ software was used for quantifying all the Western blot images. First, the scanned or digital image of the Western blot was imported into ImageJ software. Using the ‘Rectangular Selection’ tool, bands of interest corresponding to target proteins and loading controls (GAPDH) were outlined and selected. The ‘Gel Analysis’ tool was then utilised to plot lane profiles and quantify band intensities. Background subtraction was performed to minimise non‐specific signals, followed by normalisation of target protein bands to loading controls. Data analysis included calculating the ratio of band intensities for each protein of interest to the corresponding loading control (relative to GAPDH). GAPDH and β‐actin were used as loading controls throughout the study. As our work was initiated several years ago during the pandemic, the availability of certain reagents including antibodies at specific times influenced our choice between the two.

### 2.7 Immunoprecipitation

Cells were scraped from culture dishes and centrifuged to pellet cells. Cell pellets were lysed on ice for 1 h in immunoprecipitation (IP) buffer (250 mM NaCl, 50 mM Tris, .5 mM EDTA and .5% NP‐40) with protease/phosphatase inhibitor added. Lysates were centrifuged to remove cell debris and the soluble portion was precleared with isotype control antibody (Santa Cruz) and rotein A/G conjugated beads (Santa Cruz) for 1 h with agitation at 4°C. Beads were pelleted by centrifugation and lysates were moved to new tubes. The lysates were then incubated with isotype or specific antibody (1:100) on ice for 1 h. Twenty microlitres of protein A/G beads was added and samples were agitated at 4°C overnight. The beads containing the immunoprecipitated proteins were pelleted by centrifugation, and the unbound portion was decanted. The beads were washed three times in IP buffer prior to processing for immunoblot analysis.

### 2.8 Immunohistochemistry and immunofluorescence

Ten‐micrometre Formalin‐Fixed, Paraffin Embedded (FFPE)Exper tissue sections were used for immunohistochemistry and immunofluorescence experiments. For dewaxing, slides were placed in xylenol for 10 min, then 100% ethanol for 10 min, then 90% ethanol for 10 min, and last 70% ethanol for 10 min. The slides were then washed in dH_2_O for 5 min. For antigen retrieval, slides were placed in antigen retrieval solution (Vector Laboratories), and the container was microwaved at high for 5 min. For permeabilisation, slides were washed with PBS/Triton (.25%, v/v; PBST) solution twice for 10 min. Slides were then blocked in 5% bovine serum albumin in PBS for 60 min at RT. Slides were then stained with the primary antibody (1:100 in PBST) and kept in the humid box overnight at RT, followed by three washes in PBST. For immunofluorescence, slides were stained with the secondary antibody (1:100 in PBST) and DAPI for 60 min at RT. Slides were washed three more times in PBST then mounted with ProLong Diamond Antifade Mountant (Invitrogen), sealed with clear nail polish, then visualised by fluorescence microscopy. For immunohistochemistry, the anti‐goat HRP‐DAB Cell and Tissue Staining Kit (R&D systems) was used.

### 2.9 Quantitative polymerase chain reaction

Trizol (Thermo Scientific, 15596018) was used to extract total RNA from cultured cells, according to the manufacturer's instructions. Complementary DNA was then produced using High‐Capacity cDNA Reverse Transcription kit (Thermo Scientific, 4368814). Real‐time quantitative polymerase chain reaction (qPCR) was performed with Fast SYBR Green Master Mix (Life Technologies) on QuantStudio 7 Flex system (Life Technologies).

The following human‐specific primers were used in this study:

MLKL forward primer: 5′‐ATCAAAGTATTCAACAACCCC‐3′
MLKL reverse primer: 5′‐GCAAATCCCAAATATACGCAA‐3′
RIPK3 forward primer: 5′‐CGGGCACACCACAGAACAT‐3′
RIPK3 reverse primer: 5′‐GTAGCACATCCCCAGCACCAC‐3′
RIPK1 forward primer: 5′‐AGAAGAAGGGAACTATTCGC‐3′
RIPK1 reverse primer: 5′‐TTCTATGGCCTCCACGAT‐3′
GAPDH forward primer: 5′‐TCCACTGGCGTCTTCACC‐3′
GAPDH reverse primer: 5′‐GGCAGAGATGATGACCCTTTT‐3′

### 2.10 Confocal immunofluorescence microscopy

Cells were cultured in 35 mm glass bottom dishes (Cellvis #D35‐10‐1.5‐N). Cells were fixed in 4% paraformaldehyde for 10 min and permeabilised with .1% Triton‐X for 10 min at RT for intracellular labelling, followed by incubation with primary antibody for 1 h at RT. When a secondary antibody was needed, cells were incubated with respective FITC‐ or Alexa Fluor 647‐conjugated secondary antibody (Sigma–Aldrich F9137 or Thermo Scientific A21244) at a dilution of 1:100 for 1 h at RT. NucBlue Live ReadyProbes Hoechst stain (Thermo Scientific R37605) was included with secondary antibody stains when applicable, according to the manufacturer's specifications. Samples were examined under LSM 710 confocal microscope (Zeiss) using a 63× oil immersion objective. The fluorescence intensity of images was calculated using ZEN software.

### 2.11 Plaque assay

Viral egress was measured using a plaque assay. Monolayers of Optn +/+ and Optn ‒/‒ cells were plated in six‐well plates and infected with the McKrae‐WT virus at MOI .1. Media were collected at different time points post‐infection and titred on Vero cells. Briefly, primary incubation of collected media was performed with Opti‐MEM (Life Technologies) for 2 h. Vero cells were then incubated with growth media containing 1% methylcellulose for 72 h followed by fixing with 100% methanol and staining with crystal violet solution. Formed plaques were counted and analysed.

### 2.12 siRNA transfection

A Dicer‐substrate short interfering RNAs (DsiRNAs) TriFECTa Kit (IDT) with predesigned siRNA molecules were used for transfections in this study. Cells were plated and grown to 50% confluency. Cells were then transfected as per the manufacturer's protocol using RNAiMAX at 1 µL/mL in OptiMEM (Thermo Fisher). Multiple concentrations for each premade siRNA molecule were tested and it was determined that OPTN siRNA 1 at 10 nM and MLKL siRNA 1 at 30 nM produced effective knockdown with minimal cell death after 48 h of transfection.

### 2.13 Statistical analysis

The data shown in the figures are means ± standard error of means (SEM). The statistical tests were performed on GraphPad Prism. Asterisks indicate significant difference: ^*^ *p* < .05, ^**^ *p* < .01, ^***^ *p* < .001 and ^****^ *p* < .0001.

## 3 RESULTS

### 3.1 MLKL is a cellular factor that promotes HSV‐1 infection in OPTN‐deficient conditions

**FIGURE 1:**
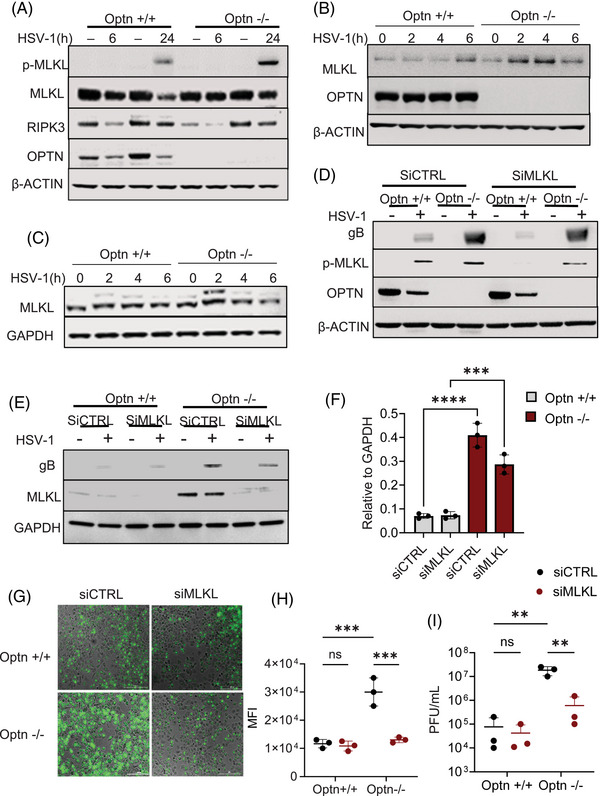
Mixed lineage kinase domain‐like (MLKL) is a cellular factor that promotes herpes simplex virus‐1 (HSV‐1) infection in Optineurin (OPTN)‐deficient conditions. (A) Representative immunoblots of Optn +/+ and Optn ‒/‒ human corneal epithelial (HCE) cells infected with HSV‐1 (.5 MOI) for 6 and 24 h. (B) Representative immunoblots of Optn +/+ and Optn ‒/‒ HCE cells exposed to 1 MOI infection for the 0, 2, 4 and 6 h post‐infection. (C) Representative immunoblots of Optn +/+ and Optn ‒/‒ HeLa cells exposed to 1 MOI infection for the 0, 2, 4 and 6 h post‐infection. (D) siCTRL and siMLKL transfected Optn +/+ and Optn ‒/‒ HCE cells were infected with 1 MOI HSV‐1 for 24 h and the total cell lysates were collected and analysed by immunoblotting against specified antibodies. (E) siCTRL and siMLKL transfected Optn +/+ and Optn ‒/‒ HeLa cells were infected with 1 MOI HSV‐1 for 24 h and the total cell lysates were collected and analysed by immunoblotting against specified antibodies. (F) Glycoprotein B (gB) expression in (E) is quantified relative to GAPDH. (G) siCTRL and siMLKL transfected Optn +/+ and Optn ‒/‒ cells were infected with 1 MOI 17 GFP‒HSV‐1 (green) for 24 h and then imaged using a fluorescent microscope. (H) Quantification of fluorescence intensity of GFP in (G). (I) siCTRL and siMLKL transfected Optn +/+ and Optn ‒/‒ cells were infected with 1 MOI HSV‐1 for 24 h and the total cell lysates were collected to determine the viral titre by a standard viral plaque assay. Data are represented as mean ± standard error of means (SEM) of triplicate samples (F, H and I), and comparable results were obtained from three independent experiments. pfu, plaque‐forming units.

Our previous work showed that OPTN‐deficient cells were likely to undergo necroptosis‐induced cell death.^5^ In consideration of the critical role MLKL plays during necroptosis, we examined the expression of MLKL and p‐MLKL (indicates activation of MLKL by RIPK3) in Optn ‒/‒ HCE cells and tissues at different stages of HSV‐1 infection. Optn +/+ and Optn ‒/‒ HCE cells were infected with HSV‐1 McKrae strain (HSV‐1) for 6 and 24 h of infection. We did not observe changes in MLKL expression in the presence or absence of OPTN at 6 h post‐infection, but there was upregulation of MLKL 24 h post‐infection in Optn ‒/‒ HCE cells (Figure 1A). Similarly, a higher expression of MLKL was also observed in HeLa cells for different HSV‐1 strains (Figure S1A‒C). We additionally noted a higher expression of p‐MLKL in Optn ‒/‒ cells after 24 h of HSV‐1 infection (Figures 1A and S7A). We also conducted a time course experiment to evaluate the expression of MLKL during the first 6 h of infection. Interestingly, we did observe a higher expression of MLKL in OPTN ‒/‒ cells at 2 and 4 h post‐infection (Figures 1B and S7B). We did not observe phosphorylation of MLKL during these early stages of HSV‐1 infection. This indicated a role of MLKL that is not dependent on its phosphorylation by RIPK3. To further confirm if the upregulation of MLKL during the early stages of infection was dependent on RIPK3, we conducted the time course experiment in a RIPK3‐deficient cell line HeLa. We observed similar upregulation of MLKL in Optn ‒/‒ cells at 2 h and 4 h post‐infection (Figures 1C and S7C). Next, we evaluated if MLKL deficiency impacts HSV‐1 infection. To analyse this, we conducted a transient knockdown of MLKL in HCE and HeLa cells. The growth of HCE or HeLa cells were not affected by knockdown of MLKL (data not shown). The accumulation of gB, an HSV‐1 envelope protein in siCTRL HCE cells, was much higher than that in siMLKL cells (Figures 1D and S7D,E). We also used 1 MOI 17 GFP‒HSV‐1 strain, a GFP‐tagged HSV‐1, to infect the cells and observed that the GFP intensity of siCTRL Optn ‒/‒ HeLa cells was much higher than that of siMLKL Optn ‒/‒ HeLa cells (Figure 1G,H). Consistently, the titre of HSV‐1 in siCTRL Optn ‒/‒ cells was also markedly higher than siMLKL Optn ‒/‒ HeLa cells infected with HSV‐1 for 24 h (Figure 1I). Collectively, these data demonstrate that upregulated MLKL aids HSV‐1 in the absence of OPTN and that the negative regulation of MLKL by OPTN constituting an MLKL‒OPTN axis is necessary to control HSV‐1 infection.

### 3.2 MLKL facilitates endosomal trafficking of HSV‐1 during the early stages of infection

**FIGURE 2:**
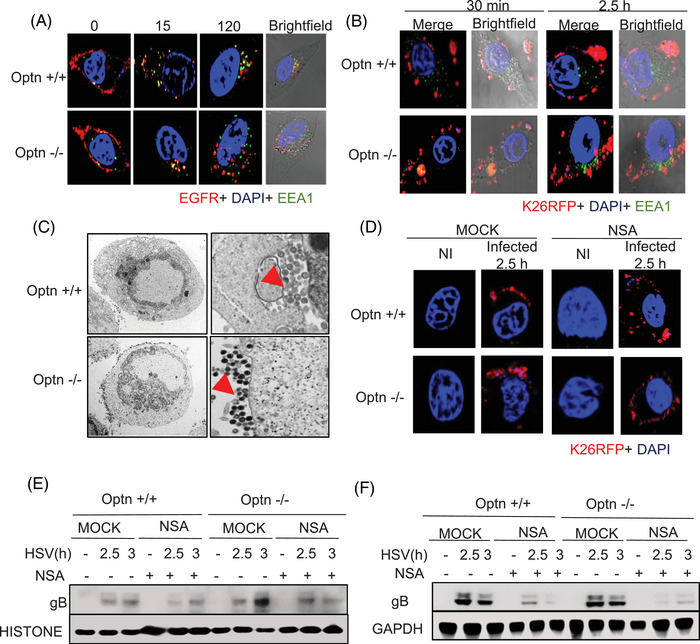
Mixed lineage kinase domain‐like (MLKL) facilitates endosomal trafficking of herpes simplex virus‐1 (HSV‐1) during the early stages of infection. (A) Optn +/+ and Optn ‒/‒ HeLa cells were incubated in serum‐free media for 12 h. These cells were then exposed to epidermal growth factor (EGF) for 30 min on ice. After being washed with PBS, cells were incubated in normal growth media with EGF at 37°C and intracellular uptake of epidermal growth factor receptor (EGFR) was monitored at indicated times by immunofluorescence microscopy. (B) Optn +/+ and Optn ‒/‒ cells were exposed to 10 MOI K26‐RFP HSV‐1 infection for 2 h on ice, followed by incubation at 37°C for 2.5 h. HSV‐1 intracellular transport was monitored by immunofluorescence microscopy. (C) Optn +/+ and Optn ‒/‒ cells were exposed to 1 MOI HSV‐1 infection for 24 h, and HSV‐1 transport to the plasma membrane was examined using transmission electron microscopy. (D) Optn +/+ and Optn ‒/‒ cells were exposed to 10 MOI K26‐RFP HSV‐1 infection for 2 h on ice, followed by incubation with or without Necrosulphonamide (NSA) (MOCK) at 37°C for 2.5 h and immunofluorescence imaging was conducted to track the transport of HSV‐1. (E and F) Optn +/+ and Optn ‒/‒ cells were exposed to 10 MOI K26‐RFP HSV‐1 infection for 2 h on ice, followed by incubation with or without NSA (MOCK) at 37°C for 2.5 and 3 h. Subcellular fractionation was conducted, and (E) nuclear fraction and (F) cytoplasmic fraction were analysed by immunoblotting. All images are representative images and similar results were obtained from three independent experiments.

Previously, we observed upregulation of MLKL during the early stages of infection (Figure 1B,C), which was independent of phosphorylation of MLKL required for canonical role of MLKL in executing necroptosis. Our imaging data also indicated an accumulation of MLKL near the nucleus at 4 h post‐infection (Figure S1D). To investigate the non‐canonical role of MLKL in the early stages of HSV‐1 infection, we conducted our next set of experiments in the RIPK3‐deficient HeLa cell lines. The initial stages of infection include viral entry followed by the transport of virions into the nucleus. Previous work already determined that OPTN is not involved in viral entry^5^; however, to our surprise, we observed a higher transport of HSV‐1 virions into the nucleus in Optn ‒/‒ cells as compared to Optn +/+ cells (Figure S2A). MLKL has been previously suggested to enhance endosomal transport so we explored the idea that the discrepancy in increased virions reaching the nucleus in the absence of OPTN was due to upregulated MLKL enhancing endocytic pathways, which may inadvertently be aiding the transport of HSV‐1 virions to the nucleus.^25^ First, we analysed if there is faster transport of molecules in Optn ‒/‒ cells in general. For this, we monitored the intracellular transport of EGFR within cells following treatment with EGF. Immunofluorescence analysis indicated that the transport of EGFR was delayed in Optn‐expressing cells with low MLKL expression in comparison to the Optn ‒/‒ cells. (Figure 2A). This faster translocation of EGFR observed in Optn ‒/‒ cells was delayed in MLKL depleted (siMLKL) Optn ‒/‒ cells (Figure S8C), further corroborating that higher expression of MLKL is instrumental in regulating the intracellular transport rate in Optn ‒/‒ cells.

A similar phenomenon was observed in HSV‐1‐infected cells where transport of HSV‐1 was delayed in Optn +/+ cells supported by our confocal imaging (Figure 2B). Our electron microscopy data showed that more HSV‐1 particles were transported to the plasma membrane in Optn ‒/‒ cells indicating higher endocytic pathway activation by MLKL co‐opted by the virus (Figure 2C). In comparison, HSV‐1 particles were still inside the cells in the Optn +/+ cells (Figure 2C). These observations corroborated our hypothesis that MLKL's enhancement of the endocytic pathway is hijacked by the virus when OPTN is deficient, and the presence of OPTN ensures curbing this MLKL‐aided viral transport of HSV‐1.

To further evaluate the importance of MLKL's modulation by OPTN in dictating endocytic pathways, we conducted experiments with a pharmacological MLKL inhibitor NSA. Immunofluorescence imaging indicated a similar rate of transport in Optn +/+ and Optn ‒/‒ cells treated with NSA (Figure 2D). Furthermore, we observed less transport of gB into the nucleus in Optn ‒/‒ cells treated with NSA in comparison to the mock‐treated Optn ‒/‒ cells (Figures 2E and S7E,F). In summary, MLKL is the driving force for enhanced transport of HSV‐1 particles in Optn ‒/‒ conditions; utilising NSA as an MLKL inhibitor specifically in OPTN‐deficient conditions helps rescue the gB levels close to that in Optn +/+, indicating unregulated MLKL's role in driving HSV‐1 transport to the nucleus. Thus, MLKL plays a critical role during early stages of HSV‐1 infection, which does not require its activation by RIPK3.

### 3.3 MLKL is selectively degraded by OPTN's ubiquitin binding domain through direct binding

**FIGURE 3:**
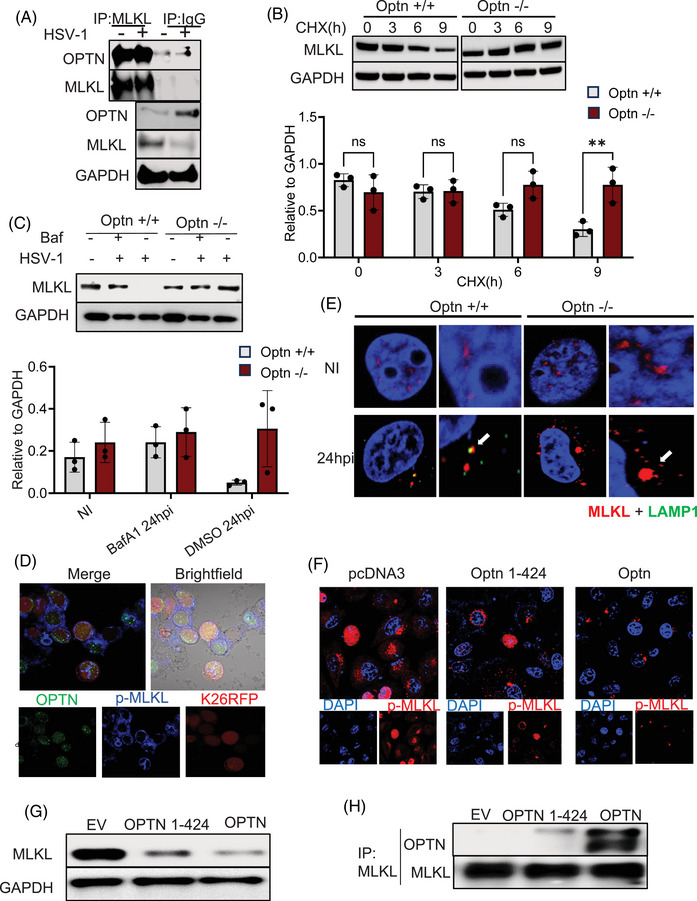
Optineurin (OPTN) utilises the ubiquitin binding domain (UBD) domain to selectively degrade mixed lineage kinase domain‐like (MLKL). (A) Optn +/+ cells were infected with or without 1 MOI herpes simplex virus‐1 (HSV‐1) for 24 h, followed by immunoprecipitation by MLKL and immunoglobulin G (IgG) (control). Total cell lysates were analysed by Western blot with indicated antibodies. (B) Optn +/+ and Optn ‒/‒ cells were subjected to 1 MOI HSV‐1 infection for 12 h. Cycloheximide (CHX) was then added, and cells were collected at the indicated time and analysed by Western blotting with indicated antibodies. (C) Optn +/+ and Optn ‒/‒ cells were subjected to 1 MOI HSV‐1 infection for 24 h with or without bafilomycin (Baf) and cell lysates were analysed by immunoblotting. (D) Optn +/+ cells were exposed to 2 MOI K26‐RFP HSV‐1 infection for 24 h and the colocalisation of Optn and p‐MLKL was observed using immunofluorescence imaging. (E) Optn +/+ and Optn ‒/‒ cells were subjected to 1 MOI HSV‐1 infection for 24 h, and MLKL and LAMP1 (a marker for lysosomal degradation) were examined with immunofluorescence imaging. The white arrows indicate colocalisation of MLKL and LAMP1. (F‒H) Optn ‒/‒ cells were transfected with empty vector control (EV), full‐length OPTN (OPTN), and OPTN 1–424 plasmids and infected with 1 MOI HSV‐1 infection for 24 h, and p‐MLKL expression was examined by (F) immunofluorescence imaging, (G) whole cell lysates were examined by immunoblotting with indicated antibodies and (H) immunoprecipitation was carried out post‐transfection and the cell lysates were immunoblotted with OPTN and MLKL to assess the interaction between the two. Data are represented as mean ± standard error of means (SEM) of triplicate samples (B and C) and similar results were obtained from three independent experiments. NI, non‐infected; WCL, whole cell lysate (WCL).

Next, we examined the mechanism by which OPTN regulates MLKL expression. As an autophagy adapter protein, OPTN is known to directly interact with the ubiquitinated cargo and carry them for autophagic degradation. In this regard, we hypothesized that the direct binding of MLKL to OPTN promotes its autophagic degradation under normal and infected conditions. To test this hypothesis, we performed a co‐IP assay by immunoprecipitating MLKL to study its potential direct interaction with OPTN. Our IP studies indicated a direct interaction between MLKL and OPTN (Figure 3A). The direct interaction between MLKL and OPTN suggested autophagic control of MLKL exerted by OPTN, and to test this, we used a cycloheximide chase assay to inhibit new protein synthesis. We found that the degradation of MLKL was OPTN‐dependent as MLKL degradation ceased in Optn ‒/‒ conditions (Figure 3B). The presence or absence of Bafilomycin (autophagy inhibitor) had no effect on MLKL degradation in Optn ‒/‒ cells (Figure 3C). In comparison, degradation of MLKL was inhibited in the presence of Bafilomycin in Optn +/+ cells (Figure 3C).

Immunofluorescence imaging shows lysosomal uptake of MLKL en route to its autophagic degradation only in the presence of OPTN indicated by the colocalisation of MLKL and Lamp1 (lysosome marker), which is abrogated in the absence of OPTN (Figure 3E). Additionally, to determine the site of interaction between MLKL and OPTN, we explored the possibility that OPTN is incorporated into the MLKL necrosome complex. Wild‐type cells were infected with 10 MOI K26‐RFP HSV‐1 infection and the intracellular location of OPTN was monitored. The results indicated that OPTN was indeed recruited to the necrosome complex (indicated by p‐MLKL rings) in response to HSV‐1 infection (Figure 3D). Furthermore, we also observed that MLKL oligomer formation was hindered in the presence of Optn (Figure S1A).

To further elucidate the novel direct interaction between MLKL and OPTN, we conducted domain mapping experiments to find the OPTN domain responsible for interaction with MLKL. Next, we pursued the idea that OPTN's ubiquitin binding domain (UBD), which is responsible for recognising cargo for autophagy, is the selective domain that interacts with and targets MLKL. To test this hypothesis, we transfected Optn ‒/‒ cells with expression vectors of full‐length OPTN and OPTN fragments lacking the UBD domain OPTN1‐424 (Figure 3F). Our results indicate that only the full‐length OPTN plasmid reduced the MLKL expression, as shown by our Western blot and imaging data, and the UBD‐deleted fragment was not successful in both binding to MLKL and sufficiently targeting it for degradation (Figure 3G,H). Collectively, our results show MLK's direct interaction with OPTN, which enables its recognition of autophagic degradation through OPTN's UBD.

### 3.4 HSV‐1 infection causes aggregation of MLKL bodies in the brains of OPTN‐deficient mice

**FIGURE 4:**
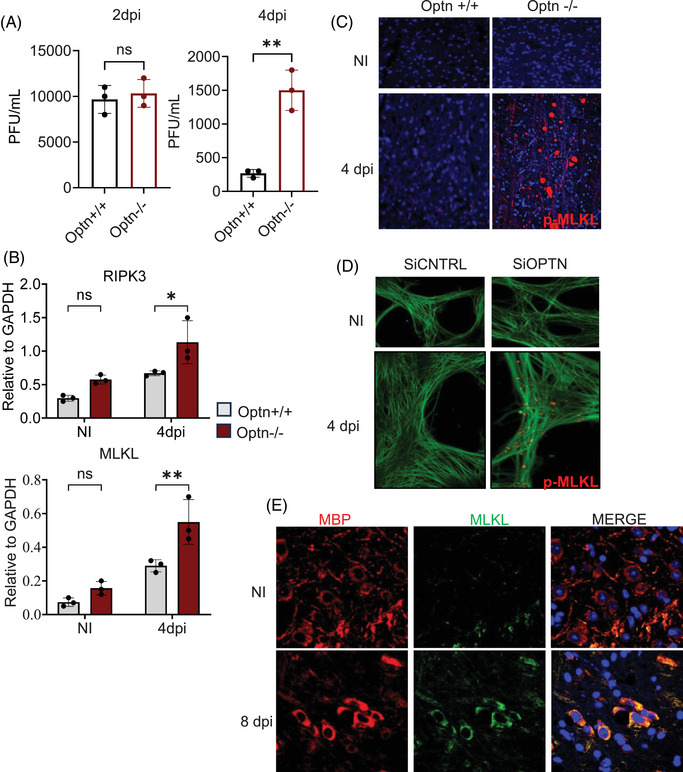
Optineurin (OPTN) restricts the formation of mixed lineage kinase domain‐like (MLKL) bodies during herpes simplex virus‐1 (HSV‐1) infection in mice. Optn +/+ and Optn −/− mice were infected with HSV‐1 (5 × 10^5^ PFU) via corneal scarification. (A) Plaque assays were conducted with corneal swabs collected 2 and 4 days post‐infection (dpi). (B) Receptor‐interacting protein kinase 3 (RIPK3) and MLKL transcripts in the brains of infected mice (harvested 4 dpi) were measured by quantitative polymerase chain reaction (qPCR) analysis. Two‐way analysis of variance (ANOVA) was performed for statistical analysis. (C) p‐MLKL expression was examined in the brainstem of infected mice (harvested 4 dpi) by immunofluorescence imaging. (D) p‐MLKL expression was examined in siCTRL and siOPTN transfected LUHMES cells infected with 2.5 MOI HSV‐1 for 24 h by immunofluorescence imaging. (E) Optn −/− mice were infected with HSV‐1 (5 × 10^5^ PFU) via corneal scarification, and colocalisation of MLKL and myelin basic protein (MBP) (a marker for oligodendrocytes) was examined in the brainstem of infected mice (harvested 8 dpi) by immunofluorescence imaging. NI, non‐infected.

To investigate whether MLKL upregulation occurs during HSV‐1 infection in vivo, we infected Optn +/+ and Optn ‒/‒ mice with ocular HSV‐1 and assessed MLKL levels in the eyes, trigeminal ganglia (TG) and brain. Consistent with our in vitro findings, Optn ‒/‒ mice exhibited higher infection rates 4 days post‐infection (dpi) (Figure 4A), with the eye serving as the primary site of HSV‐1 infection. In line with the data presented in Figure 1, elevated MLKL protein expression was observed in whole‐eye lysates from Optn ‒/‒ mice 4 dpi (Figure S3A). Additionally, we noted increased MLKL, RIPK3 and RIPK1 transcripts in eye lysates and TG of HSV‐1‐infected Optn ‒/‒ mice compared to Optn +/+ mice (Figure S4A‒D). Given HSV‐1's neurotropic nature, we assessed MLKL activation in the mouse brain, which revealed significantly higher RIPK3 and MLKL transcripts (Figure 4B). To understand the previously unexplored consequences of this significant activation of MLKL in brain tissue exacerbated by the loss of OPTN in response to HSV‐1 infection, we used immunofluorescence imaging that revealed extensive MLKL and p‐MLKL aggregates (referred to as MLKL bodies) in the brainstem of only Optn ‒/‒ mice 4 dpi (Figures 4C and S3B). This aggregation of MLKL bodies also occurred when we silenced OPTN in HSV‐1‐infected human mesencephalic neuronal cells (LUHMES), which suggested that MLKL was disposed to form aggregates (MLKL bodies) in neuronal cells and tissue in the absence of OPTN (Figure 4D).

To further understand the impact of these MLKL bodies in vivo, we evaluated the expression of different cell types in the brain in Optn +/+ and Optn ‒/‒ mice during HSV‐1 infection (Figure S5). We observed a significant decrease in the expression of oligodendrocytes in Optn ‒/‒ mice brains during HSV‐1 infection, as shown in our imaging data (Figures S5 and 5A). Myelin basic protein (MBP) is the second most abundant protein in the myelin accounting for 30% of the total CNS myelin protein. It is synthesised in the CNS oligodendrocytes and essential for myelin formation and its long‐term maintenance. The pathogenic role of MBP is associated with MS where it could act as an autoantigen, inducing self‐attack on myelin sheath, leading to demyelination. So MBP was used as a biomarker to evaluate expression of Oligodendrocytes. To assess the hypothesis that the decrease in oligodendrocytes driven by HSV‐1 infection specifically occurring in Optn ‒/‒ mice is due to the aggregation of MLKL bodies that are driving necroptotic cell death in oligodendrocytes, we utilised immunohistochemistry. Our experiments revealed colocalisation of MBP and MLKL in HSV‐1‐infected brainstems of Optn ‒/‒ mice, corroborating the role of MLKL in the death of these oligodendrocytes during HSV‐1 infection (Figure 4E). Taken together, we show evidence of extensive loss of oligodendrocytes as a consequence of HSV‐1 infection in the brainstem of Optn ‒/‒ mice driven primarily by the aggregation of catastrophic MLKL bodies.

**FIGURE 5:**
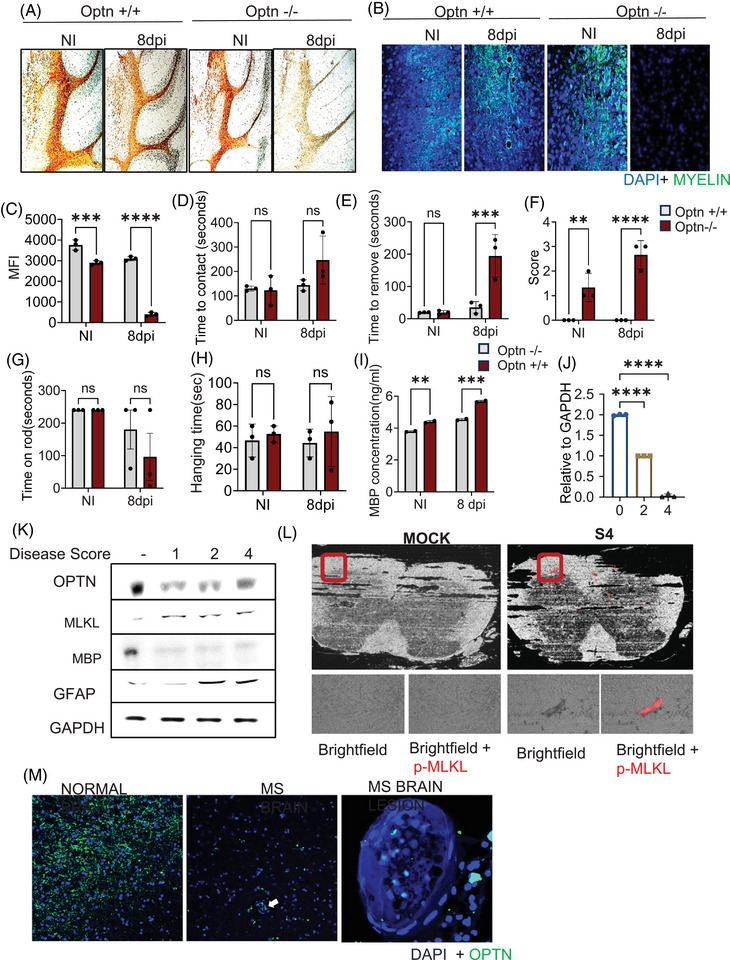
Optineurin (OPTN) deficiency displays a phenotype similar to demyelinating disorders. Optn +/+ and Optn −/− mice were exposed to no infection and herpes simplex virus‐1 (HSV‐1; 5 × 10^5^ PFU) via corneal scarification. (A) Immunohistochemistry was performed to analyse myelin basic protein (MBP) expression in the cerebellum of non‐infected and infected mice (harvested 8 days post‐infection [dpi]). (B) Immunofluorescence was used to analyse myelin expression in the brainstem of mice indicated by FluoroMyelin (green). (C) Quantification of fluorescence intensity of FluoroMyelin from (B). (D and E) A tape removal test was performed on infected and non‐infected mice, and the time to contact (recognise the tape) and time to remove the tape was noted. (F) Ledge test was performed on infected and non‐infected mice and scores were noted. (G) Rotarod test was performed on infected and non‐infected mice and the time the mice took to stay on the rod without falling was monitored. (H) Wire test was performed on infected and not infected mice and the time it took to hold the wire before falling was noted. (I) MBP concentration in serum was measured by ELISA. (J) Optn transcript was measured in the brains of EAE mice by quantitative polymerase chain reaction (qPCR). 0, 1, 2 and 4 are EAE mice disease progression scores. Refer to the methods section for scoring details. (K) Brain lysates from EAE mice indicate protein levels of MBP, GFAP, mixed lineage kinase domain‐like (MLKL) and OPTN. 0, 1, 2 and 4 are EAE mice disease progression scores. Refer to the methods section for scoring details. (L) p‐MLKL expression was monitored in the spinal cord tissues of EAE mice. S4 is disease progression score 4 for EAE mice. (M) Optn expression levels were examined in the normal brain tissues and multiple sclerosis (MS) brain tissues. The white arrow indicates the MS brain lesion. Data are presented as mean values  ± standard error of means (SEM) (C‒I). Two‐way analysis of variance (ANOVA) was performed for statistical analysis (C‒I). Two‐tailed Student's *t*‐test was performed for statistical analysis (J). NI, non‐infected.

### 3.5 MLKL dysregulation shows MS‐like motor defects in HSV‐1‐infected mice

Upregulated MLKL in the Optn ‒/‒ mice post‐HSV‐1 infection‐induced oligodendrocyte death, specifically in the brainstem. Oligodendrocytes are cells responsible for producing the protective myelin sheath around neurons and play crucial roles in maintaining neuronal homeostasis. We proceeded to evaluate the effect of MLKL‐driven oligodendrocyte death by assessing demyelination in these tissues. As hypothesized, we observed reduced myelin expression in the brains of Optn ‒/‒ mice (Figure 5B). Remarkably, oligodendrocyte death and myelin loss were specifically localised to the brain stem and cerebellum regions (Figure 5A,B). The cerebellum plays a critical role in coordinating voluntary movements, maintaining balance, and fine‐tuning motor skills, contributing to the precision and smoothness of physical activities. To determine the effect of MLKL‐induced oligodendrocyte death on the coordination and balance of mice after HSV‐1 infection, we conducted a series of behavioural studies on Optn +/+ and Optn ‒/‒ mice. Initially, we performed the tape removal test to assess sensorimotor coordination. Before infection, both Optn +/+ and Optn ‒/‒ mice could sense and remove the tape at similar times. However, after HSV‐1 infection, Optn ‒/‒ mice took significantly longer to remove the tape, indicating a substantial coordination defect (Figure 5D,E). Subsequently, to evaluate balance problems, we utilised the ledge test and observed that Optn ‒/‒ mice displayed shaking behaviour and reluctance to lower themselves into the cage, resembling cerebellar ataxia in humans (Figure 5F). The rotarod test, which examines motor skills, and the grip test, did not yield statistically significant results (Figure 5G,H).

Demyelination induced by unregulated MLKL in Optn ‒/‒ mice contributed extensively to significant defects that bore a resemblance to demyelinating disorders. Demyelination typically involves the shedding of myelin, which can be detected in the serum of patients. To evaluate myelin shedding in our model, we measured the levels of shedding MBP in mouse serum. HSV‐1‐infected Optn ‒/‒ mice displayed the highest levels of MBP in the serum, supporting our hypothesis of severe demyelination occurring due to dysregulated MLKL and MBP shedding compared to the Optn +/+ mice (Figure 5I). The series of behaviour tests we performed on mice highlighted impairments that were phenotypically similar to the extensively studied demyelinating disorder MS, often studied using the EAE mouse model. Brain lysates from EAE mice showed similar reductions in mRNA and protein levels of OPTN, which indicated that there may be a dysregulated MLKL pathway activation in these tissues as well (Figures 5J,K and S7G). Additionally, increasing MLKL and GFAP (a marker for CNS injury) protein levels alongside decreasing MBP, corresponding to the increasing disease score for EAE mice depicting the MS state, reinforced the dysregulation of the MLKL‒OPTN axis.

Strikingly, we also found higher levels of MLKL and RIPK3 transcripts in these EAE mice (Figure S4E). The spinal cord is frequently affected in MS, causing motor, sensory and autonomic dysfunction. Interestingly, similar to the brains of our HSV‐1‐infected Optn ‒/‒ mice, we detected aggregates of MLKL bodies in the spinal cords of these EAE mice (Figure 5L). Lastly, we also observed lower OPTN levels in brain tissues of MS lesions collected from humans compared to the normal counterparts (Figure 5M). Overall, the EAE mice with progressively worse MS disease exhibited similar increases in MLKL and loss of MBP and OPTN that was like changes we report in Optn‐deficient HSV‐1‐infected mice. Collectively, these data suggested that the intricate link between MLKL and OPTN that serves to maintain myelin, with dysregulation of this balance resulting in severe impairments in motor functions and behaviours reported typically in MS and MS‐like demyelinating disorders.

### 3.6 NSA treatment rescues the MLKL‐induced demyelination during HSV‐1

**FIGURE 6:**
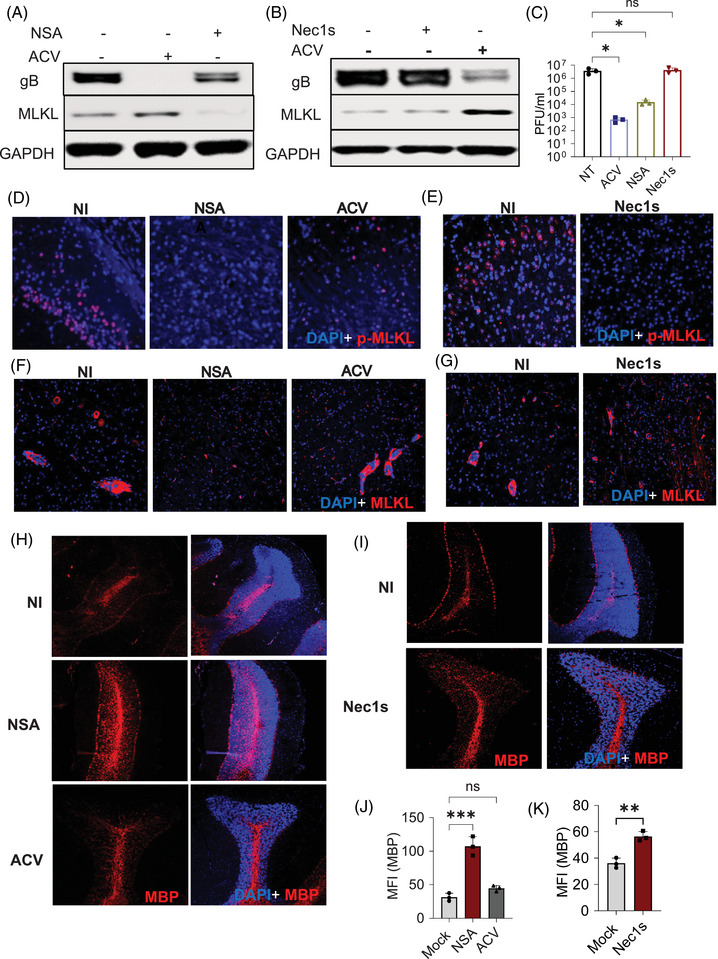
Pharmacological inhibition of mixed lineage kinase domain‐like (MLKL) is required to prevent demyelination during Herpes simplex virus‐1 (HSV‐1) infection in Optn‐deficient conditions. (A) Optn ‒/‒ cells were infected with 1 MOI HSV‐1 and treated with DMSO (control), Necrosulphonamide (NSA) and Acyclovir (ACV) for 24 h, and cell lysates were analysed by immunoblotting with indicated antibodies. (B) Optn ‒/‒ cells were infected with 1 MOI HSV‐1 and treated with DMSO (control), Necrostatin 1s (Nec1s) and ACV for 24 h, and cell lysates were analysed by immunoblotting with indicated antibodies. (C) Optn ‒/‒ cells were infected with 1 MOI HSV‐1 and treated with DMSO (control), NSA, Nec1s and ACV for 24 h, and plaque assay was performed. (D, F and H) Optn ‒/‒ mice were infected with HSV‐1 (5 × 10^5^ PFU) via corneal scarification. After 4 days post‐infection (dpi), the mice were treated with two daily doses of DMSO (control), NSA and ACV till 8 dpi. Brainstem slides were probed for (D) p‐MLKL, (E) MLKL and the cerebellum slides were probed for (H) myelin basic protein (MBP) by immunofluorescence imaging. (E, G and I) Optn ‒/‒ mice were infected with HSV‐1 (5 × 10^5^ PFU) via corneal scarification. After 4 dpi, the mice were treated with two daily doses of DMSO (control) and Nec1s till 8 dpi. Brainstem slides were probed for (E) p‐MLKL, (J) MLKL and cerebellum slides were probed for (I) MBP by immunofluorescence imaging. (J and K) Quantification of MBP fluorescence intensity in (H) and (I) respectively. One‐way analysis of variance (ANOVA) was used for statistical analysis (C, J and K).

Since the upregulation of MLKL leads to demyelination in Optn ‒/‒ mice during HSV‐1 infection, we investigated if inhibiting MLKL using NSA could mitigate demyelination in these mice. Our initial in vitro studies suggested that NSA treatment reduced MLKL expression in Optn ‒/‒ cells during HSV‐1 infection (Figures 6A and S6B). Next, we conducted in vivo studies, and like the in vitro studies, we observed a reduction in MLKL and p‐MLKL expression with NSA treatment in Optn ‒/‒ and Optn +/+ mice brains (Figures 6D,F and S6F). Consecutively, our in vivo study also revealed that NSA treatment rescued the loss of myelin in Optn ‒/‒ infected mice (Figure 6H). To elucidate if MLKL expression regulation in Optn ‒/‒ is dependent on higher HSV‐1 titres, we conducted in vitro and in vivo experiments with Acyclovir (ACV), which is the standard of care therapy to treat herpes patients. Interestingly, the reduction of virus by ACV did not reduce the MLKL and p‐MLKL expression levels (Figure S6A,D,F and S8A). ACV treatment was also unsuccessful in rescuing the damage phenotype of oligodendrocytes and demyelination defects associated with increased MLKL suggesting that standalone ACV therapy in response to virus‐induced MLKL activation and upregulation is insufficient and while the initial trigger in the pathology may have been the virus, the tight regulation of the MLKL‒OPTN axis protects against this severe neuropathy (Figure 6H,J).

To further clarify if demyelination in these mice was a result of the cell death‐dependent role of MLKL, we performed in vitro and in vivo studies with Nec1s, which is a known necroptosis inhibitor that inhibits phosphorylation of MLKL required to execute necroptosis. Nec1s treatment was able to reduce phosphorylation of MLKL but there was no change in basal levels of MLKL both in vitro and in vivo (Figure 6B,E,G, S8B). Also, there was minimal MBP expression rescue in Nec1s‐treated mice but not as significant as compared to NSA treatment (Figure 6I,K). Together, these data indicated that reduction of p‐MLKL is not sufficient to reduce demyelination, and there might be a cell death‐independent role of MLKL acting in conjunction that contributes to the demyelination we see.

## 4 DISCUSSION

HSV‐1 infected over half of the global population, yet reported clinical cases remain disproportionately scarce compared to its widespread seroprevalence. This discrepancy underscores the complex interplay between host defenses and viral evasion mechanisms, offering a rich terrain for exploration into the epidemiology of HSV‐1.^26^ Cell death pathways, particularly necroptosis, have emerged as critical components of host defense against viral infections. Previous studies have identified strategies HSV‐1 utilises to evade this tightly regulated cell death; however, little is known of the tight regulation of the necroptotic pathway and its execution against HSV‐1, particularly the molecular players associated with regulating the pathway and the consequences of the rampant hyperactivation of MLKL‐mediated cell death.^27^ The diverse non‐necroptotic functions of MLKL, which may be upregulated in response to infection, also warrant deeper exploration.

Here, we identify OPTN as a sentinel molecule exerting its control on MLKL and regulating its functions within the host's cellular machinery, beyond its previously identified role as a host restriction factor against HSV‐1. In this study, we show that OPTN plays a pivotal role in the negative regulation of MLKL by directly binding and targeting it for autophagic degradation. This regulatory role emerges as a critical function during HSV‐1 infection as we demonstrate that the absence of OPTN and the subsequent destabilisation of the MLKL‒OPTN axis results in promoting hyperactivated MLKL's catastrophic cell death functions and non‐necroptotic roles.^25^, ^28^ We showed that dysregulated MLKL assists in the endosomal trafficking of HSV‐1 virions to the nucleus, inadvertently benefiting the replication of the virus and providing novel compelling evidence of hyperactivated MLKL causing widespread neuronal damage by targeting oligodendrocytes through the accumulation of MLKL bodies, which contributes to extensive demyelination of the neurons of infected animals, causing severe impairments mimicking those occurring in MS‐like disorders.^14^, ^29^

Our results indicated that MLKL's roles in endosomal trafficking facilitate nuclear transport of HSV‐1, suggesting a previously unreported role for OPTN in controlling HSV‐1 entry into the nucleus via endocytosis. When the MLKL‒OPTN axis is disrupted by the loss of OPTN, endocytic pathways may be more primed to carry viruses without autophagic regulation of endosomal compartments and their cargo. Further work is required to determine whether Optn deficiency can also enhance other types of HSV‐1 intracellular transport, like transport of HSV‐1 glycoproteins to assembly sites, through MLKL‐dependent and independent pathways.^30^, ^31^

Neurotropic herpesviruses, particularly HSV have been the subject of scientific investigation for a long time, particularly in their associations with neurodegenerative conditions such as Alzheimer's disease.^32^, ^33^ In the current study, our aim was to unravel host factors that drive neuronal damage during HSV‐1 infection that may be priming the molecular landscape of the central nervous system towards disease processes such as demyelinating disorders and in that regard show the disruption of the MLKL‒OPTN axis in driving widespread demyelination resulting in behavioural and functional deficits in our animal model. Our NSA experiments also suggest supplementation of MLKL inhibitors with standard‐of‐care ACV therapy may be necessary to prevent oligodendrocyte damage associated with MLKL activity; however, further study into this necessary.^34^ NSA treatment also contributed to a better rescue of oligodendrocytes compared to Nec‐1s indicating another potential non‐necroptotic function of MLKL in the destruction of the myelin sheath. MLKL undergoes phosphorylation at T357, S358 and S360 during necroptosis activation, while studies suggest a cell death‐independent activation by phosphorylation at S441, involved in myelin sheath breakdown, which our in vitro results corroborate.^11^, ^35^, ^36^ We show increased expression of phospho‐MLKL S441 in Optn ‒/‒ cells suggesting that this non‐necroptotic role may come to the forefront in a disrupted MLKL‒OPTN axis. In the current study, we were impeded in our pursuit of this function by the unavailability of a commercially available p‐MLKL S441 antibody that is reactive against mice and emphasises future investigation of this observation.

The findings in this study align with and expand upon the emerging understanding of MLKL as a multifunctional protein with roles extending beyond its canonical function in necroptosis. Traditionally recognised for its terminal role in executing programmed necrotic cell death through membrane permeabilisation,^37^ MLKL has also been implicated in endosomal sorting^25^ and cell adhesion regulation.^38^ Our study demonstrates that in the context of HSV‐1 infection, MLKL's endosomal trafficking functions become hijacked by the virus, leading to enhanced nuclear delivery of viral particles. This suggests that MLKL's engagement in intracellular transport pathways, when unregulated, may inadvertently facilitate viral replication for HSV‐1. Moreover, in this study, we also highlight the necroptotic role of MLKL, which gets activated during the later stages of the infection leading to death of oligodendrocytes. Thus, our data integrated MLKL's diverse cellular functions into a coherent model in which its dysregulation drives both necroptotic and non‐necroptotic pathology during neuroinvasive HSV‐1 infection.

The MLKL mechanism uncovered in HSV‐1 infection may extend to other ocular and neurotropic pathogens that exploit vesicular trafficking for replication and spread, such as VZV, CMV and rabies virus. These pathogens similarly rely on intracellular transport, suggesting that MLKL dysregulation—particularly in the absence of OPTN—could facilitate their dissemination and contribute to tissue damage. This indicates the role of MLKL‒OPTN axis as a broader regulatory hub with potential relevance beyond HSV‐1, where its disruption may drive neuroinflammation or demyelination in diverse infectious contexts. However, further studies are needed to determine whether similar MLKL‐mediated mechanisms are at play in these other infections.

Finally, demyelinating disorders remain a huge burden on healthcare and the global population.^39^ Our observations of demyelinating disorder‐like behaviours and pathology caused by dysregulated MLKL, along with studies using EAE mice to investigate shared molecular signatures, were particularly revealing. Interestingly, these analyses uncovered reduced OPTN expression levels in the MS disease mouse model.^35^, ^40^ These EAE mice like our Optn‐deficient mice displayed higher levels of RIPK3 and MLKL.^40^ Human MS brain tissue samples also showed a lower expression of OPTN.

Altogether these results underscore the broad impact of MLKL‒OPTN axis and how its dysregulation may not only contribute to various pathologies but may be the invisible chain between herpesviruses infections and MLKL‐driven demyelination. This represents a novel mechanism where during HSV‐1 infection, absence of OPTN leads to hyperactivation of MLKL indicated with the formation of MLKL bodies. This hyperactivation drives the death of oligodendrocytes resulting in demyelination and leading to MS‐like pathologies. This establishes a molecular connection between HSV‐1 and demyelinating diseases.

We demonstrate the crucial role of the MLKL‒OPTN axis in adequately puppeteering MLKL's functions, both necroptotic and non‐cell death‐related.^29^ By elucidating the molecular intricacies of the MLKL‒OPTN axis, our findings not only provide insights into HSV‐1 pathogenesis but also offer potential therapeutic targets for mitigating viral‐induced neuronal damage, paving the way for future investigations into novel therapeutic strategies and our understanding of viral pathogenesis.

## 5 CONCLUSION

In conclusion, this study highlights the non‐necroptotic role of MLKL, which is unfolded in the absence of OPTN, enhancing the endosomal trafficking of HSV‐1 virions to the cell nucleus. This process leads to severe demyelination of neurons and oligodendrocytes, ultimately causing cell death. Additionally, treatment with a MLKL inhibitor demonstrated therapeutic potential by preserving the myelin sheath and reducing neurologic damage during HSV‐1 pathogenesis.

## AUTHOR CONTRIBUTIONS

*Conceptualisation and investigation*: Ilina Bhattacharya. *Methodology*: Ilina Bhattacharya, Tejabhiram Yadavalli, Chandrashekhar D. Patil, Rashmi Kadam, Ipsita Volety, Sergey Kalinin and Douglas L. Feinstein. *Writing—original draft*: Ilina Bhattacharya. *Writing—review and editing*: Ilina Bhattacharya, Rashmi Kadam, Ipsita Volety and Deepak Shukla. *Mouse model development*: Henry C. Tseng. *Funding acquisition*: Deepak Shukla. *Supervision*: Deepak Shukla.

## CONFLICT OF INTEREST STATEMENT

The authors declare they have no conflicts of interest.

## ETHICS STATEMENT

All experiments were conducted in compliance with the regulations of the University of Illinois at Chicago. All animal procedures were conducted in accordance with institutional guidelines and approved protocols to ensure humane treatment of mice.

## Supporting information




**Figure S1**: Altered MLKL expression post HSV‐1 infection. (A) Representative immunoblots of Optn +/+ and Optn ‒/‒ cells exposed to 1 MOI HSV‐1 infection for 24h. (B) Quantification of MLKL in (A) relative to GAPDH. (C) Representative immunoblots of Optn +/+ and Optn ‒/‒ cells exposed to 0.1MOI and 1MOI infection with three different HSV‐1 strains: 17gfp, McCrae, and KOS for 24h. (D) Representative images of Optn +/+ and Optn ‒/‒ cells exposed to 10 MOI HSV‐1 infection and cells were fixed and imaged at 30min, 1 h, 2 h and 4 h after infection.
Figure **S2**: MLKL aids endocytic transport of HSV‐1 into the nucleus. (A) Optn +/+ and Optn ‒/‒ cells were exposed to 10 MOI HSV‐1 infection, and cells were fixed and imaged at 2 h post‐infection. (B) Optn +/+ and Optn ‒/‒ cells were exposed to 10 MOI K26‐RFP HSV‐1 infection for 2 h on ice, followed by incubation at 370C for 2.5h. HSV‐1 intracellular transport was monitored by immunofluorescence microscopy. (C) Intracellular uptake of EGFR was monitored at indicated times by immunofluorescence microscopy. (D) Optn +/+ and Optn ‒/‒ cells were exposed to 10 MOI K26‐RFP HSV‐1 infection for 2 h on ice, followed by incubation with or without NSA at 370C for 2.5 h and immunofluorescence imaging was conducted to track the transport of HSV‐1. (E) Optn +/+ and Optn ‒/‒ cells were subjected to 1 MOI HSV‐1 infection for 24h, and MLKL and LAMP1 (a marker for phagolysosomal degradation) were examined with immunofluorescence imaging. (F) Optn ‒/‒ cells were transfected with Full‐length OPTN (OPTN) and OPTN 1‐424(OPTN lacking UBD region), and cell lysates were blotted with two different OPTN antibodies.
**Figure S3**: (A) Optn +/+ and Optn ‒/‒ mice were infected with HSV‐1 (5 × 10^5^ PFU) via corneal scarification and whole eye cell lysates were analyzed with immunoblotting. (B) MLKL expression was examined in brainstem of infected mice (harvested 4 days after infection) by immunofluorescence imaging (C) Optn +/+ mice were infected with HSV‐1 (5 × 10^5^ PFU) via corneal scarification and colocalization of MLKL and MBP (marker for oligodendrocytes) was examined in brainstem of infected mice (harvested 8 days after infection) by immunofluorescence imaging.
**Figure S4**: Optn deficiency leads to upregulation of necroptosis in tissues. Optn +/+ and Optn ‒/‒ mice were infected with HSV‐1 (5 × 10^5^ PFU) via corneal scarification and transcript levels for indicated genes were analyzed in the following tissues 8 days post‐infection using qPCR: (A) non‐infected eye of the mice, (B) infected eye of the mice, (C) non‐infected TG of the mice, (D) infected TG of the mice. (E) RIPK3 and MLKL transcript was measured in brains of EAE mice by qPCR.
Figure S5: Expression of different cell types in the brain during HSV‐1 infection. Optn +/+ and Optn ‒/‒ mice were infected with HSV‐1 (5 × 10^5^ PFU) via corneal scarification, and brain tissue was collected 8 days post‐infection. Immunohistochemistry (IHC) was performed in these tissues using (A) MBP for oligodendrocytes, (B) NeuN for neurons, (C) GFAP for astrocytes, and (D) Iba1 for microglia. All these images A‐D are from the brainstem region. (E) Representative image for MBP IHC staining in the frontal cortex region of the brain.
Figure S6: Pharmacological intervention to inhibit MLKL during HSV‐1 infection. (A) Representative images of Optn +/+ and Optn ‒/‒ mice eyes infected with HSV‐1 (5 × 10^5^ PFU) via corneal scarification, followed by treatment with two daily doses of DMSO (control), NSA, and ACV from 4dpi till 8 dpi. (B) Optn +/+ cells were infected with 1 MOI HSV‐1 and treated with DMSO (control), NSA, and ACV for 24 h, and cell lysates were analyzed by immunoblotting with indicated antibodies. (C) Optn +/+ cells were infected with 1 MOI HSV‐1 and treated with DMSO (control), Nec1s, and ACV for 24 h, and cell lysates were analyzed by immunoblotting with indicated antibodies. (D) Optn +/+ cells were infected with 1 MOI HSV‐1 and treated with DMSO (control), NSA, and ACV for 24 h, and a plaque assay was performed. (E) Optn +/+ cells were infected with 1 MOI HSV‐1 and treated with DMSO (control), Nec1s, and ACV for 24 h, and a plaque assay was performed. (F) Optn +/+ mice were infected with HSV‐1 (5 × 10^5^ PFU) via corneal scarification. After 4 days post‐infection, the mice were treated with two daily doses of DMSO (control), NSA, and ACV till 8 days post‐infection. Brain slides were probed for p‐MLKL and MLKL.
Figure S7: (A–D) Quantification of western blot from Figure 1A–1D. (E–F) Quantification of western blot from Figure 2E and 2F. (G) Quantification of western blot from Figure 5K.
**Figure S8**: (A–B) Quantification of western blot from Figure 6A and 6B. (C) Confocal imaging for Optn +/+ and Optn ‒/‒ HeLa cell line knocked down for MLKL to indicate endosomal trafficking using EGF as a stimulus at 0 and 15 mins.
