# Supplementary material for: MLKL‒OPTN axis regulates herpesvirus‐induced neurological sequelae
Source: Clin Transl Med. 2025 Jun 9;15(6):e70353. doi: 10.1002/ctm2.70353 (PMC12148954; doi:10.1002/ctm2.70353)
Supplement: Supplementary file 1 — Figure S1: Altered MLKL expression post HSV‐1 infection. (A) Representative immunoblots of Optn +/+ and Optn ‒/‒ cells exposed to 1 MOI HSV‐1 infection for 24h. (B) Quantification of MLKL in (A) relative to GAPDH. (C) Representative immunoblots of Optn +/+ and Optn ‒/‒ cells exposed to 0.1MOI and 1MOI infection with three different HSV‐1 strains: 17gfp, McCrae, and KOS for 24h. (D) Representative images of Optn +/+ and Optn ‒/‒ cells exposed to 10 MOI HSV‐1 infection and cells were fixed and imaged at 30min, 1 h, 2 h and 4 h after infection. Figure S2: MLKL aids endocytic transport of HSV‐1 into the nucleus. (A) Optn +/+ and Optn ‒/‒ cells were exposed to 10 MOI HSV‐1 infection, and cells were fixed and imaged at 2 h post‐infection. (B) Optn +/+ and Optn ‒/‒ cells were exposed to 10 MOI K26‐RFP HSV‐1 infection for 2 h on ice, followed by incubation at 370C for 2.5h. HSV‐1 intracellular transport was monitored by immunofluorescence microscopy. (C) Intracellular uptake of EGFR was monitored at indicated times by immunofluorescence microscopy. (D) Optn +/+ and Optn ‒/‒ cells were exposed to 10 MOI K26‐RFP HSV‐1 infection for 2 h on ice, followed by incubation with or without NSA at 370C for 2.5 h and immunofluorescence imaging was conducted to track the transport of HSV‐1. (E) Optn +/+ and Optn ‒/‒ cells were subjected to 1 MOI HSV‐1 infection for 24h, and MLKL and LAMP1 (a marker for phagolysosomal degradation) were examined with immunofluorescence imaging. (F) Optn ‒/‒ cells were transfected with Full‐length OPTN (OPTN) and OPTN 1‐424(OPTN lacking UBD region), and cell lysates were blotted with two different OPTN antibodies. Figure S3: (A) Optn +/+ and Optn ‒/‒ mice were infected with HSV‐1 (5 × 10^5^ PFU) via corneal scarification and whole eye cell lysates were analyzed with immunoblotting. (B) MLKL expression was examined in brainstem of infected mice (harvested 4 days after infection) by immunofluorescence imaging (C) Optn +/+ mice were infected with HSV‐1 (5 × 10^5^ [file CTM2-15-e70353-s001.pptx]

## Slide 1
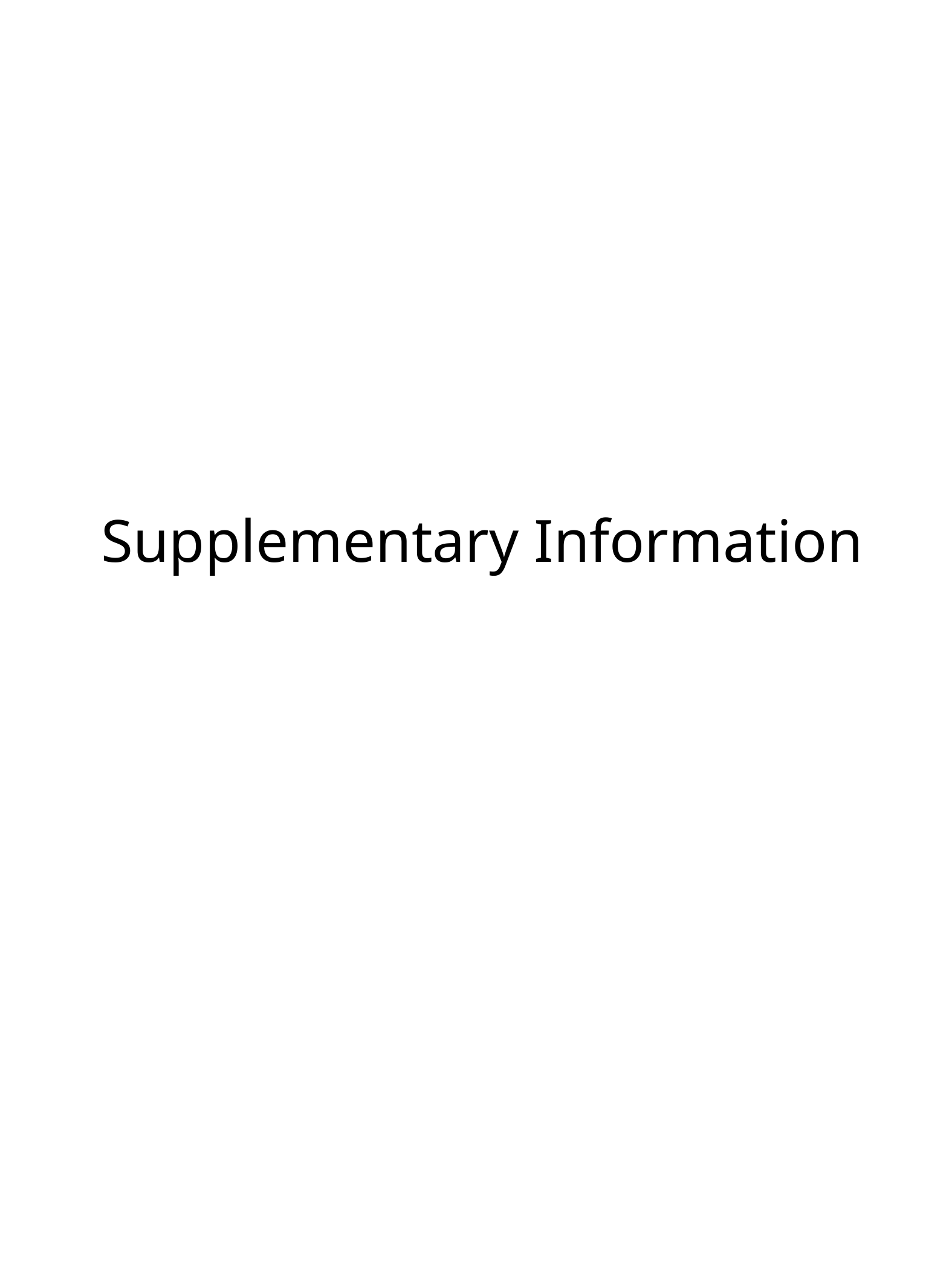

# Supplementary Information

## Slide 2
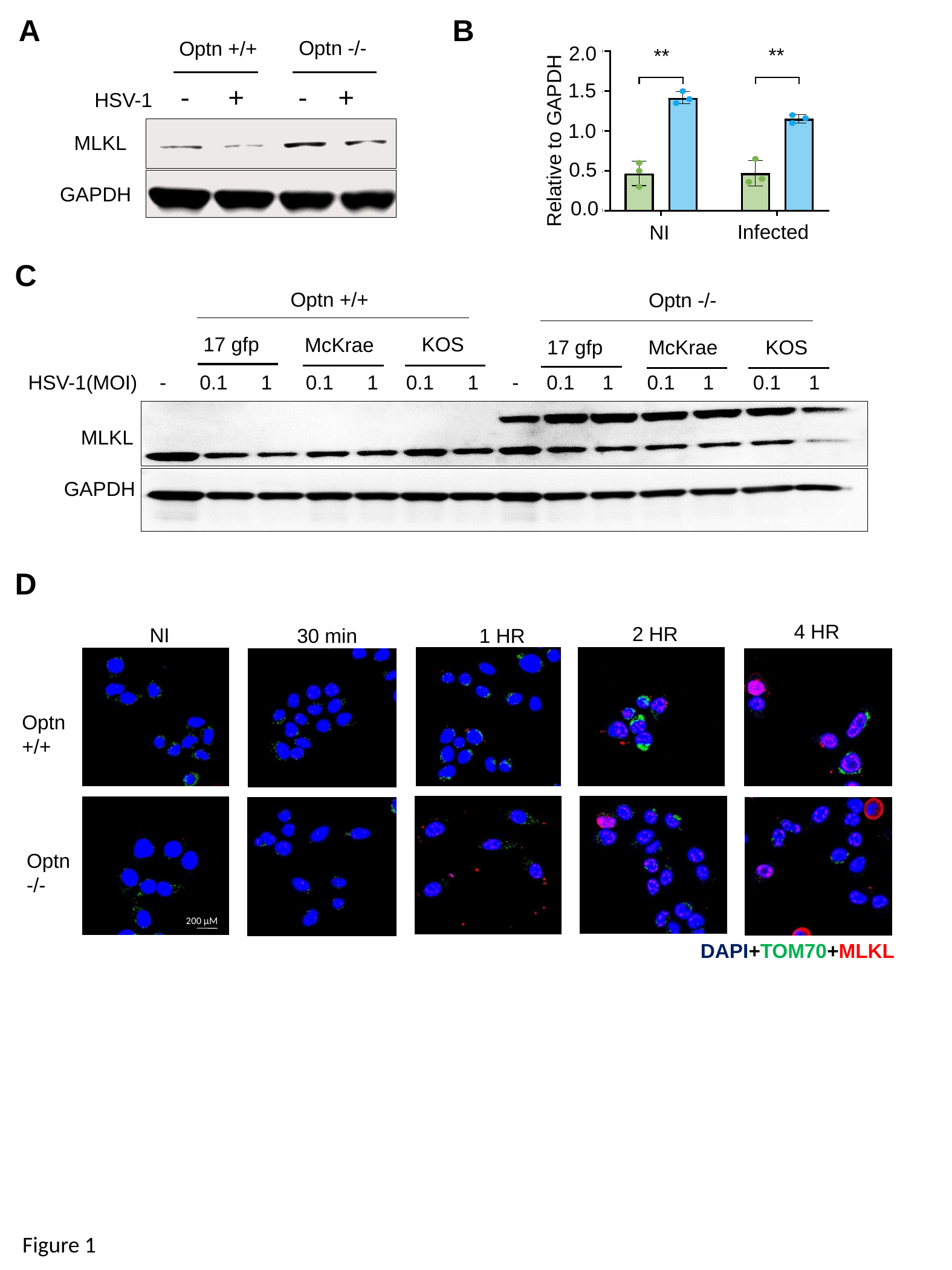

A
B
2.0
1.5
1.0
Relative to GAPDH
0.5
0.0
Infected
NI
**
**
Optn -/-
Optn +/+
MLKL
GAPDH
HSV-1 - + - +
C
Optn +/+
17 gfp
KOS
McKrae
Optn -/-
17 gfp
KOS
McKrae
HSV-1(MOI) - 0.1 1 0.1 1 0.1 1 - 0.1 1 0.1 1 0.1 1
MLKL
GAPDH
D
4 HR
2 HR
NI
1 HR
Optn +/+
Optn -/-
DAPI+TOM70+MLKL
30 min
200 µM
Figure 1

## Slide 3
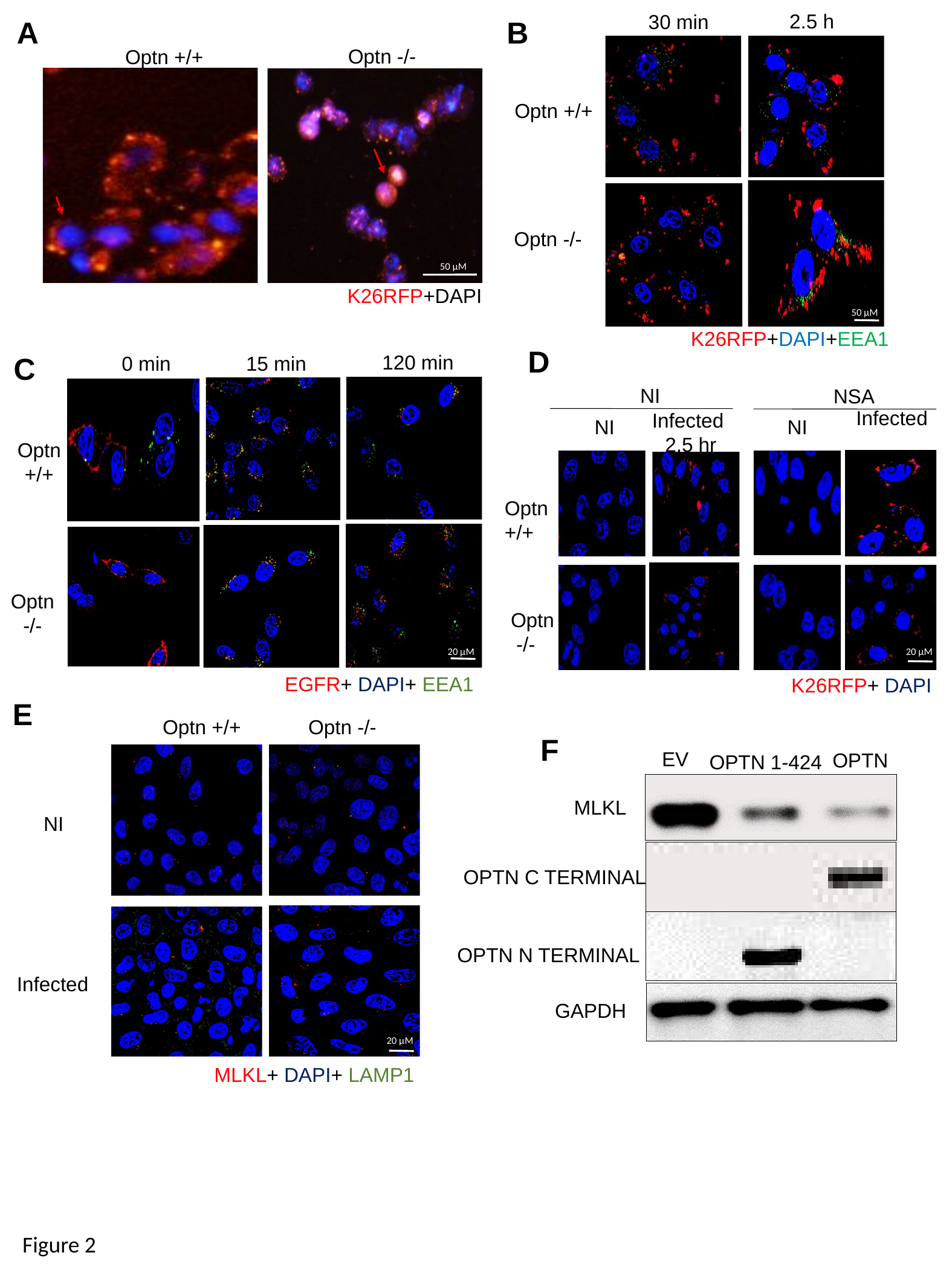

2.5 h
30 min
B
A
Optn -/-
Optn +/+
Optn +/+
C
Optn -/-
50 µM
K26RFP+DAPI
50 µM
K26RFP+DAPI+EEA1
D
120 min
15 min
0 min
Optn
+/+
Optn-/-
C
NI
NSA
Infected
2.5 h
Infected 2.5 hr
NI
NI
Optn +/+
Optn
 -/-
20 µM
20 µM
EGFR+ DAPI+ EEA1
K26RFP+ DAPI
E
Optn +/+
Optn -/-
NI
Infected
F
EV
 OPTN
 OPTN 1-424
MLKL
GAPDH
OPTN C TERMINAL
OPTN N TERMINAL
20 µM
MLKL+ DAPI+ LAMP1
Figure 2

## Slide 4
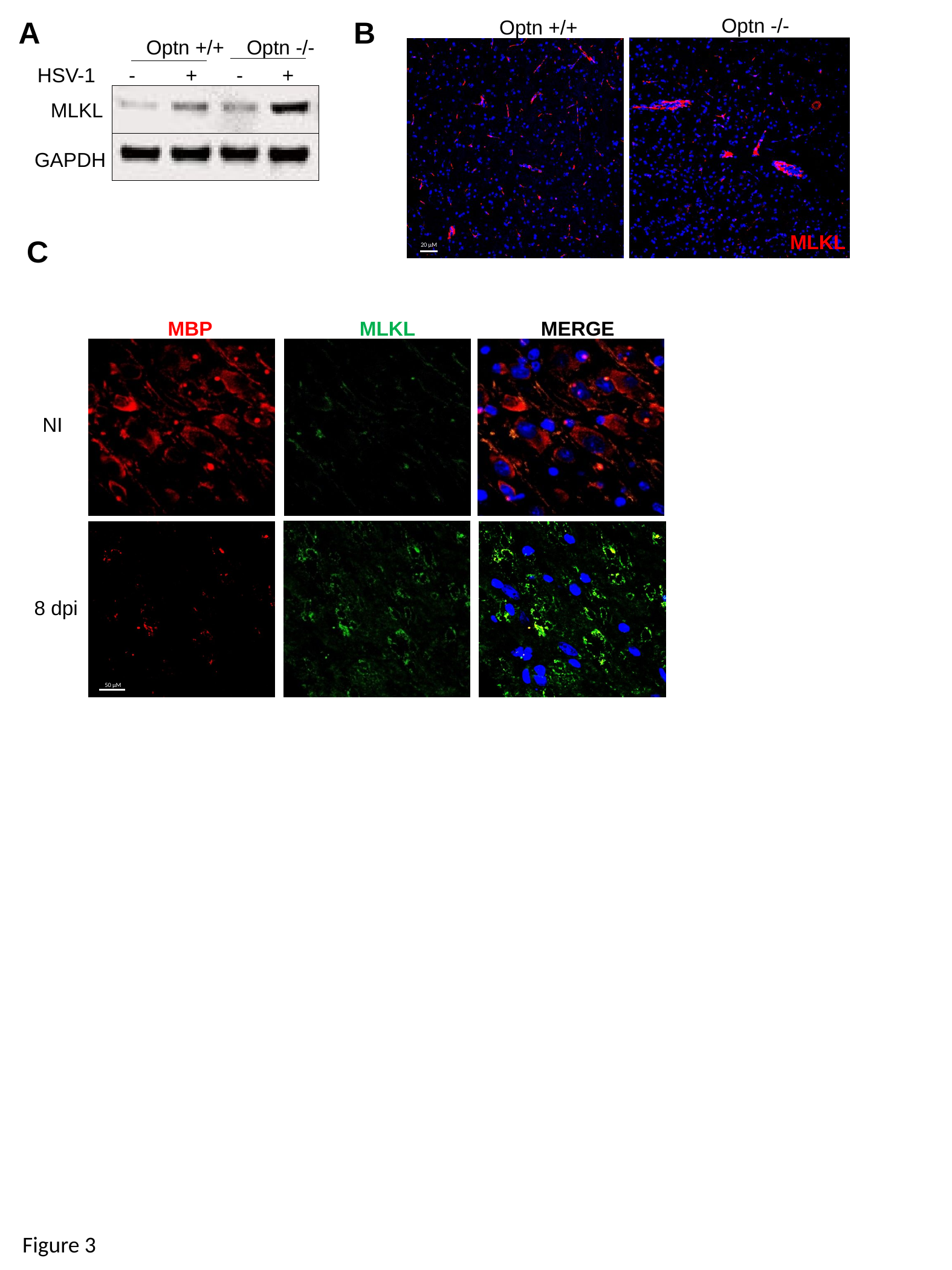

Optn -/-
Optn +/+
MLKL
A
B
Optn -/-
Optn +/+
HSV-1 - + - +
MLKL
GAPDH
C
20 µM
MLKL
MERGE
MBP
NI
8 dpi
50 µM
Figure 3

## Slide 5
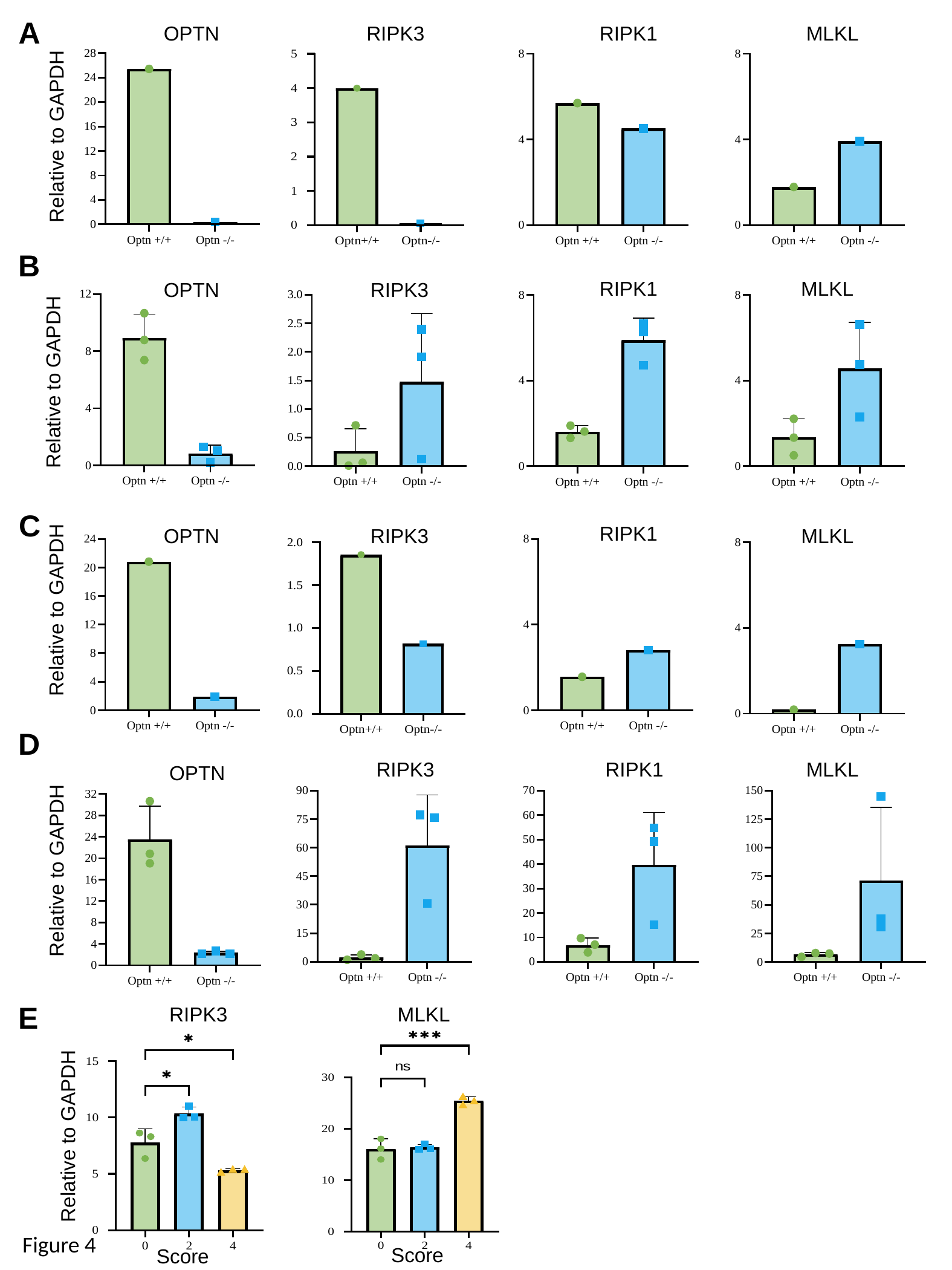

A
OPTN
RIPK3
RIPK1
MLKL
Relative to GAPDH
B
RIPK1
MLKL
OPTN
RIPK3
Relative to GAPDH
C
RIPK1
OPTN
RIPK3
MLKL
Relative to GAPDH
D
RIPK3
RIPK1
MLKL
OPTN
Relative to GAPDH
E
RIPK3
MLKL
Relative to GAPDH
Figure 4
Score
Score

## Slide 6
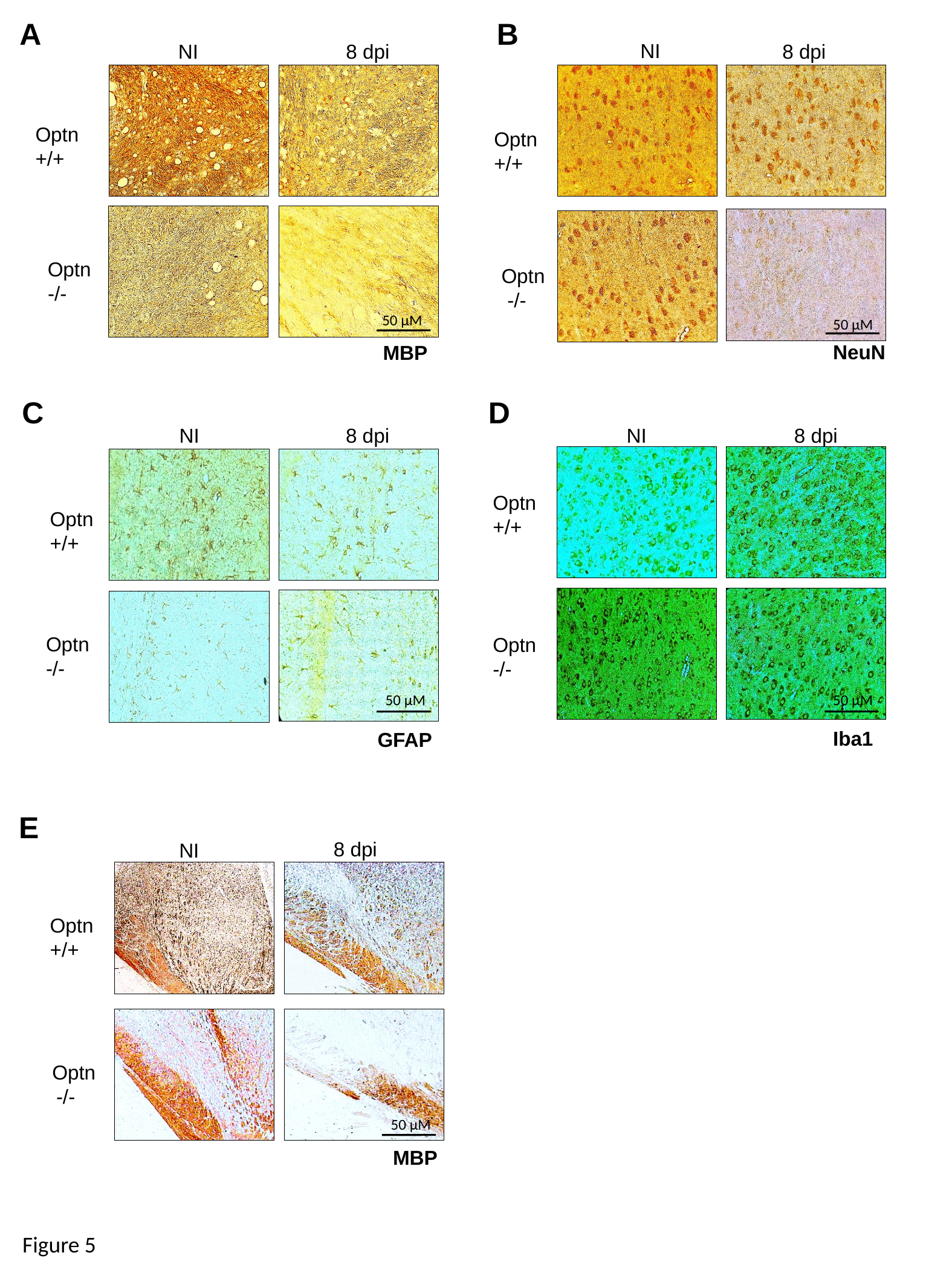

A
B
NI
8 dpi
Optn
+/+
Optn
 -/-
NeuN
NI
8 dpi
Optn+/+
Optn
 -/-
50 µM
50 µM
MBP
C
D
NI
8 dpi
Optn+/+
Optn
-/-
NI
8 dpi
Optn
+/+
Optn
-/-
50 µM
50 µM
Iba1
GFAP
E
8 dpi
NI
Optn+/+
Optn
 -/-
MBP
50 µM
Figure 5

## Slide 7
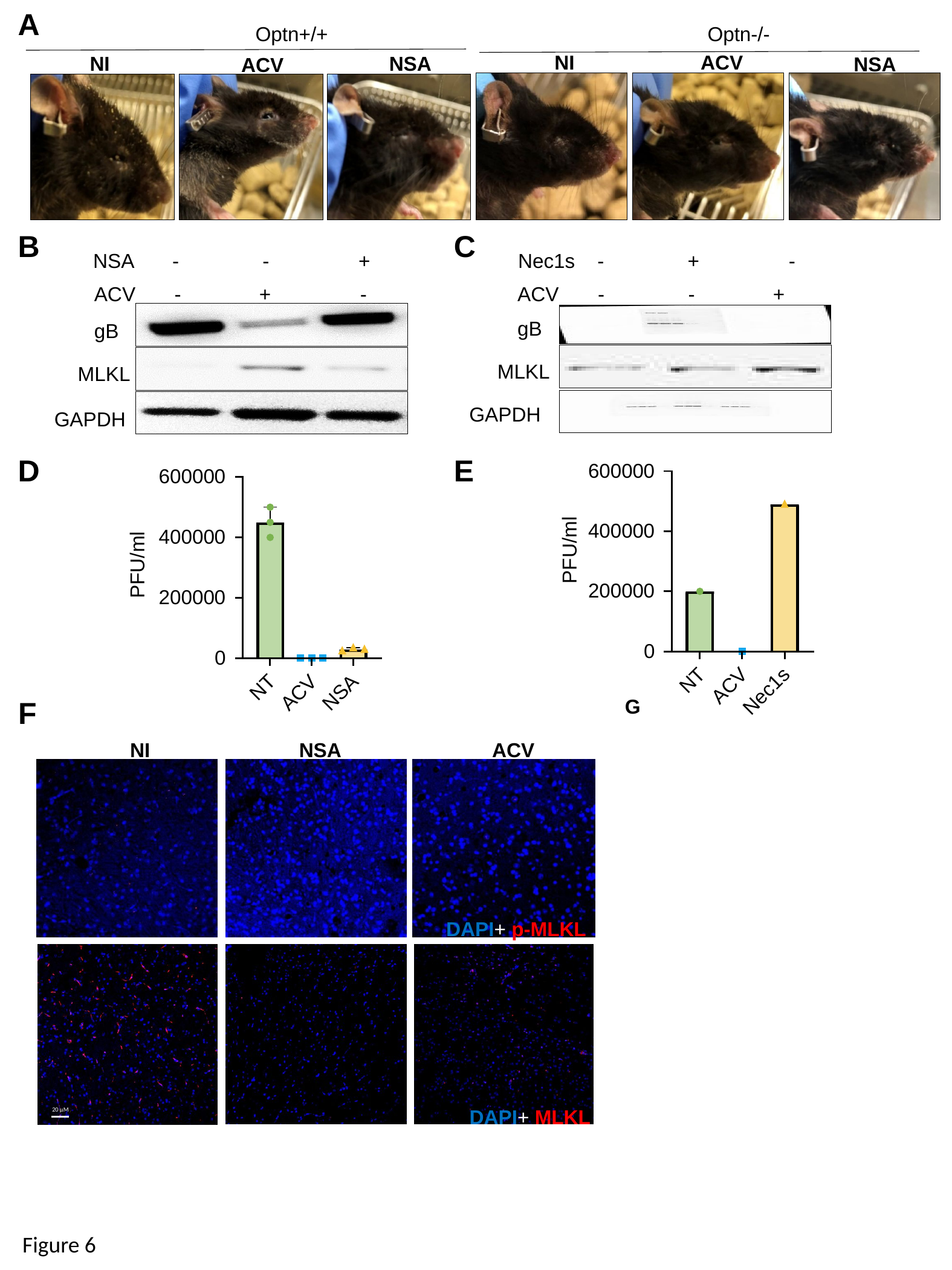

A
Optn+/+
Optn-/-
NI
ACV
NI
NSA
NSA
ACV
B
C
NSA - - +
ACV - + -
gB
MLKL
GAPDH
Nec1s - + -
ACV - - +
gB
MLKL
GAPDH
PFU/ml
PFU/ml
E
D
G
F
ACV
NI
NSA
DAPI+ p-MLKL
DAPI+ MLKL
20 µM
Figure 6

## Slide 8
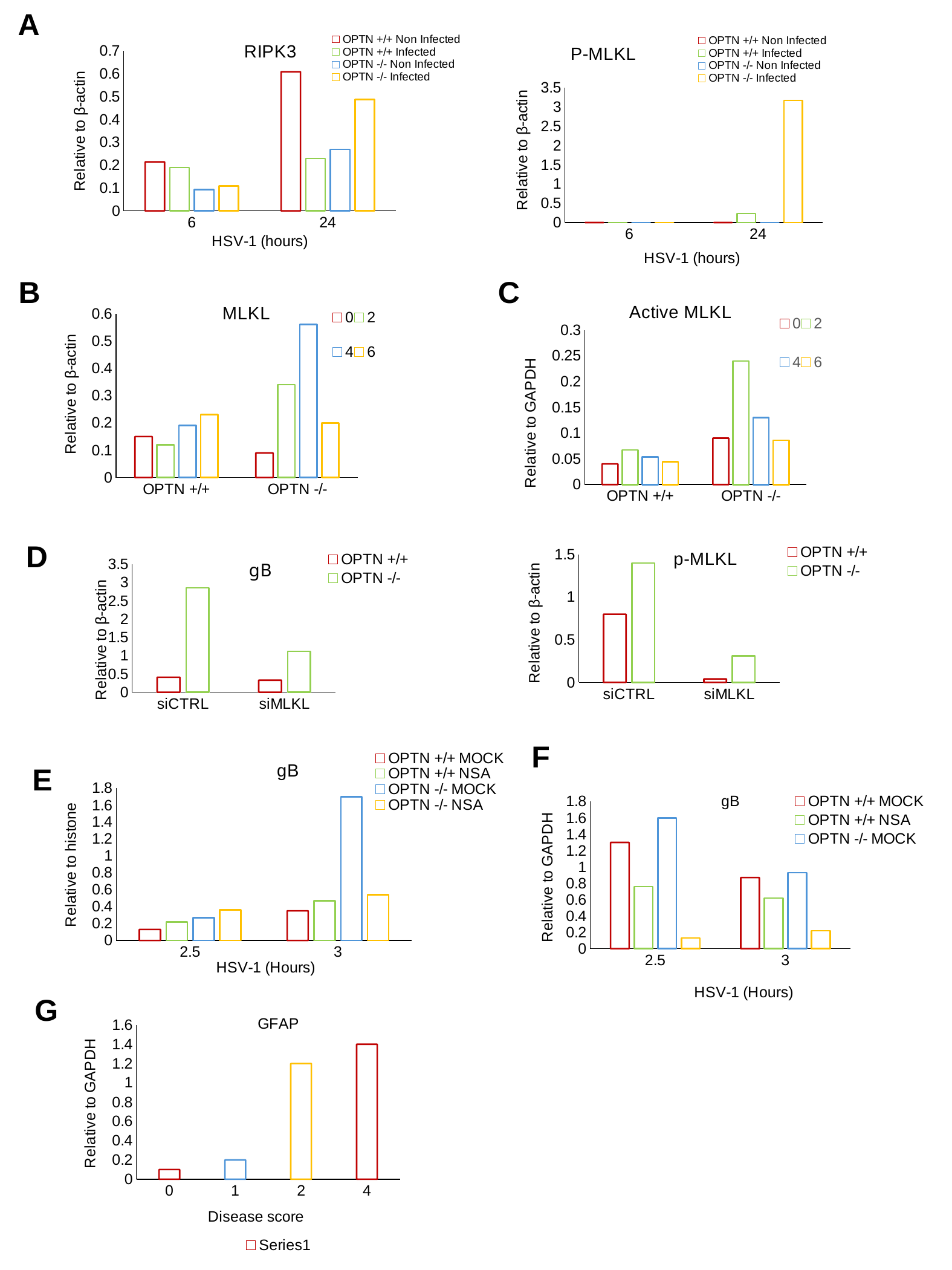

A
### Chart: RIPK3
| Category | OPTN +/+ | OPTN +/+ | OPTN -/- | OPTN -/- |
|---|---|---|---|---|
| 6 | 0.2142639526 | 0.1896999708 | 0.09346836768 | 0.1091026768 |
| 24 | 0.6090198083 | 0.2290458803 | 0.2688907818 | 0.4876058331 |
### Chart: P-MLKL
| Category | OPTN +/+ | OPTN +/+ | OPTN -/- | OPTN -/- |
|---|---|---|---|---|
| 6 | 0.0 | 0.0 | 0.0 | 0.0 |
| 24 | 0.0 | 0.2361303622 | 0.0 | 3.17 |B
C
### Chart: Active MLKL
| Category | 0 | 2 | 4 | 6 |
|---|---|---|---|---|
| OPTN +/+ | 0.04 | 0.067 | 0.054 | 0.044 |
| OPTN -/- | 0.09 | 0.24 | 0.13 | 0.086 |
### Chart: MLKL
| Category | 0 | 2 | 4 | 6 |
|---|---|---|---|---|
| OPTN +/+ | 0.15 | 0.12 | 0.19 | 0.23 |
| OPTN -/- | 0.09 | 0.34 | 0.56 | 0.2 |D
### Chart: p-MLKL
| Category | OPTN +/+ | OPTN -/- |
|---|---|---|
| siCTRL | 0.8 | 1.4 |
| siMLKL | 0.04 | 0.31 |
### Chart: gB
| Category | OPTN +/+ | OPTN -/- |
|---|---|---|
| siCTRL | 0.41 | 2.86 |
| siMLKL | 0.33 | 1.12 |F
### Chart: gB
| Category | OPTN +/+ | OPTN +/+ | OPTN -/- | OPTN -/- |
|---|---|---|---|---|
| 2.5 | 0.13 | 0.22 | 0.27 | 0.36 |
| 3 | 0.35 | 0.47 | 1.7 | 0.54 |E
### Chart: gB
| Category | OPTN +/+ | OPTN +/+ | OPTN -/- | OPTN -/- |
|---|---|---|---|---|
| 2.5 | 1.3 | 0.76 | 1.6 | 0.13 |
| 3 | 0.87 | 0.62 | 0.93 | 0.22 |G
### Chart: GFAP
| Category | |
|---|---|
| 0 | 0.1 |
| 1 | 0.2 |
| 2 | 1.2 |
| 4 | 1.4 |

## Slide 9
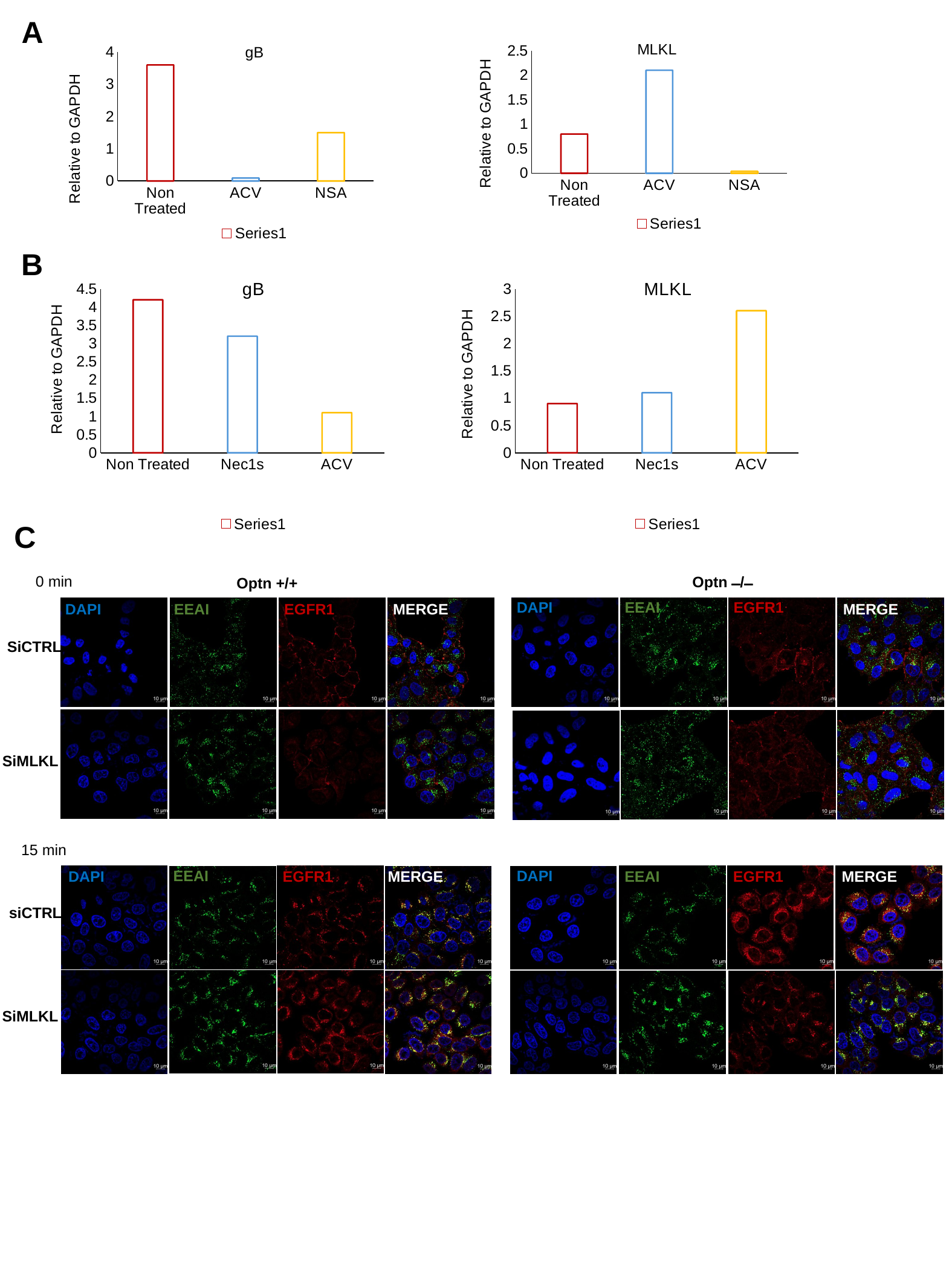

A
### Chart: gB
| Category | |
|---|---|
| Non Treated | 3.6 |
| ACV | 0.09 |
| NSA | 1.5 |
### Chart: MLKL
| Category | |
|---|---|
| Non Treated | 0.8 |
| ACV | 2.1 |
| NSA | 0.04 |B
### Chart: gB
| Category | |
|---|---|
| Non Treated | 4.2 |
| Nec1s | 3.2 |
| ACV | 1.1 |
### Chart: MLKL
| Category | |
|---|---|
| Non Treated | 0.9 |
| Nec1s | 1.1 |
| ACV | 2.6 |C
0 min
Optn ̶̶̶ / ̶̶̶
Optn +/+
EEAI
EGFR1
DAPI
MERGE
MERGE
EGFR1
DAPI
EEAI
SiCTRL
SiMLKL
15 min
DAPI
EGFR1
EEAI
MERGE
MERGE
EGFR1
DAPI
siCTRL
SiMLKL
EEAI
